# Corals Ba/Ca records uncover mid-twentieth century onset of land use change associated with industrial deforestation in Malaysian Borneo

**DOI:** 10.1038/s41598-025-06679-2

**Published:** 2025-07-01

**Authors:** Walid Naciri, Arnoud Boom, Nicola Browne, Noreen J. Evans, Kai Rankenburg, Bradley J. McDonald, Ramasamy Nagarajan, Jennifer McIlwain, Jens Zinke

**Affiliations:** 1 University of Leicester, 1 University Road, Leicester, LE1 7RH UK; 2 Molecular and Life Sciences, Curtin University, Bentley, WA 6102 Australia; 3 School of the Environment, University of Queensland, Brisbane, QLD 4072 Australia; 4 John de Laeter Centre, Curtin University, Bentley, WA 6102 Australia; 5 Department of Applied Sciences (Applied Geology), Curtin University Malaysia, 98009 Miri, Malaysia; 6 Curtin Malaysia Research Institute, Curtin University Malaysia, 98009 Miri, Malaysia; 7 Collections and Research Centre, West Australian Museum, Welshpool, WA 6106 Australia

**Keywords:** Corals, Ba/Ca, Proxy, Deforestation, Southeast Asia, Element cycles, Environmental impact

## Abstract

The increasing demand for wood, pasture, and palm oil drives deforestation and stands as the largest threats to rainforests. Whilst many consequences of deforestation are well understood, the effects on coastal ecosystems remain less clear. This issue is very apparent in Malaysian Borneo where the lack of historical deforestation data makes characterising baseline environmental conditions challenging. Building upon a previous study testing the suitability of coral Ba/Ca records as proxies for riverine sediment, we extend these records to the late nineteenth century, revealing a significant mid-20th-century surge in riverine barium levels, and a gradual lag within records consistent with distance from the river. We argue this increase is associated with the onset of industrial deforestation supported by historical logging records as well as land use data. Ba/Ca records provide unequivocal evidence for the temporal onset and magnitude of the impact of deforestation raising baseline sediment discharge in the nearshore waters.

## Introduction

In order to support the continued growth and economic development of a growing human population, forests have been cleared to create larger pastures, increase food production, or utilise wood as a valuable resource. As such, reliable estimates of tropical forested areas worldwide totalled 17 million km^2^ in 1990 compared to 15 million km^2^ in 2020^1^. Despite such a drastic observation, deforestation rates have not decreased, remaining stable during the last decades^2^. The impacts are enhanced in the tropics due to the multiple functions and roles tropical forests play, in addition to absorbing atmospheric carbon, and hosting tremendous biodiversity^3^. Indeed, tropical forests play a crucial role in local and regional precipitation due to processes such as evapotranspiration, as evidenced by several studies^4,5^. Further, during evapotranspiration, tropical forests dissipate latent heat, resulting in lower temperatures compared to those observed following deforestation^6,7^. Finally, because of their impact on precipitation, and soil and water retention^8,9^, tropical forests also regulate local and regional river runoff^10,11^. Some key tropical regions in the world are experiencing ongoing high deforestation rates, such as the Amazonian^12^ and Borneo forests^1^. The latter have been subjected to increasingly high deforestation rates during recent decades which resulted in an overall decline in forested area of 2.4 million ha (Table S1) from 1973 to 2015^13,15^.

While tracking deforestation trends has been possible using satellite imagery (Table S1), there remains a notable gap in deforestation records prior to the satellite imagery era (i.e. pre-1973) which has limited our understanding of the effects on local river runoff and sediment discharge. As such, the extent and inception of a sediment discharge increase due to deforestation have yet to be uncovered. Filling these information gaps is necessary in a biodiversity hotspot such as Borneo^16,17^ in order to better understand future trajectories under sustained deforestation. As such, characterising the baseline of sediment input into the coastal system and defining when deforestation actually begins to impact sediment discharge are critical.

In a previous study, we used sediment in river waters (as recorded by massive coral Ba/Ca ratios) off the coast of Malaysian Borneo to study the impact of increasing deforestation and associated land use change on the local coastal system over the past three decades. Indeed, deforestation was shown to lead to an increase in river discharge^18^. The results of this study were supported by an earlier sediment trap study across the Miri-Sibuti Coral Reefs National Park (MSCRNP) that revealed sustained sediment delivery to coral reefs and a coral community composition reflecting turbid reef environments^19^.

The use of geochemical archives such as corals with calcium carbonate skeletons can palliate the lack of instrumental data prior to the satellite era^20^. Based on the assumption that some trace elements can replace calcium in the aragonite lattice of the coral skeleton, it is possible to reconstruct the seawater trace element composition at the time of coral precipitation by measuring the geochemical composition of the skeleton^21,22^.

The Ba atom is one such trace element incorporated into the coral skeletal lattice^23^. Most of the Ba found in the coastal environment is believed to have a continental origin, enabling the Ba/Ca ratio in coral skeletons to serve as a historical indicator of the sediment content in river discharge^24–26^. Here, based on previous studies linking increased soil erosion with deforestation in the catchment^27^, as well as increased Ba concentrations in seawater^18^ and on land^28^ with decreasing distance from the river mouth, as well as results validating our method in this region^18^, we will look beyond the reliable observational record of regional climate and deforestation using coral proxies of sediment discharge to test the hypothesis that Ba/Ca ratios can uncover the onset of deforestation and its impact on sedimentation ultimately arriving in the Miri–Sibuti Coral Reef National Park over the past century.

## Results

### Coral Ba/Ca records of sediment in river discharge

**Fig. 1:**
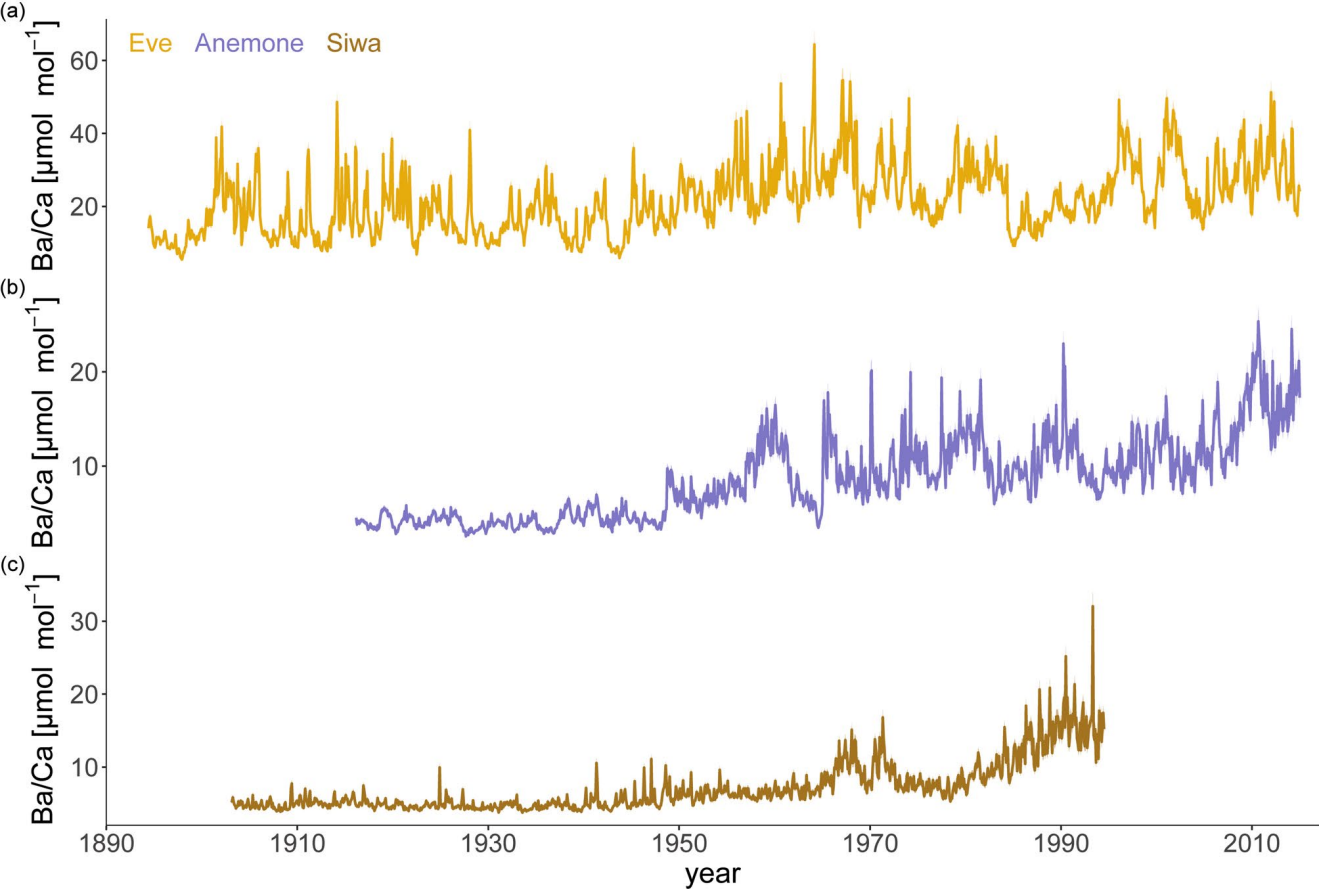
Monthly interpolated time series of (**a**) reconstructed Ba/Ca for Eve’s Garden in yellow, (**b**) Anemone Garden in blue, and (**c**) Siwa in brown. Shading indicates analytical error.

We developed Ba/Ca records for three coral cores within the Miri–Sibuti Coral Reef National Park (MSCRNP) from three separate reefs at varying distances from the main river sources of sediment and freshwater, namely the nearshore platform reef Eve’s Garden (Eve), further offshore Anemone’s Garden (Anemone), and Siwa. All three Ba/Ca records showed statistically significant different means when comparing Anemone and Eve, Anemone and Siwa, and Eve and Siwa (p < 0.001 for all three Mann–Whitney U tests). Eve showed the highest average values at 21.64 µmol mol^−1^ (Fig. 1a) compared to Anemone (8.57 µmol mol^−1^, Fig. 1b) and Siwa (7.08 µmol mol^−1^, Fig. 1c). Temporally, the Siwa and Anemone cores showed moderately low and consistent Ba/Ca values from the early 1900’s to the late 1960’s after which levels showed considerable variability. Interestingly, there has been a rapid and recent increase in Ba/Ca levels observed at Anemone in the last 5 years (2009–2014). By contrast, the coral core from Eve returned both the highest average values and greatest variability of Ba/Ca (between 5 and 60 µmol mol^−1^) with a mid-twentieth century baseline jump in Ba/Ca.

Anemone and Eve showed a weak agreement across Anemone’s (the shortest record) length on annual scales (r = 0.3057, p = 0.0022, N = 99). Conversely, neither Eve nor Anemone showed any agreement with Siwa (p = 0.31 and p = 0.28, respectively). For the Siwa core, there was a short-lived decrease in the Ba/Ca values around the year 1994 (see Fig. 1c) which happened at the position of a break between the core slab sections. Therefore, all data more recent than this break (1994 to 2015) was discarded and not used in correlation analysis to avoid overinterpretation. Correlations with river discharge were not performed as the record only starts in 1989.

### Relationship of Ba/Ca with local rainfall and river discharge

When correlated against precipitation, Anemone, Eve and the composite record C1 did not show significant results on annual scales, although correlations involving Eve were significant at the 90 % level (r = 0.1547, p = 0.0901, N = 121), while Siwa showed a significant, but weak, negative correlation (r = − 0.23, p = 0.0237, N = 91). When using a shortened version of the precipitation record (i.e. from 1950 or 1970), all three records show correlation improvements (Eve: r = 0.32, p = 0.0098, N = 65 and r = 0.3814, p = 0.0101, N = 45, respectively), although not always significant to the 95 % confidence interval (e.g. Anemone: r = 0.2448, p = 0.1051, N = 45, and Siwa: r = − 0.384, p = 0.0591, N = 25). As mentioned previously, the Baram River discharge record (Fig. S1) length is quite short (1989 to 2015) compared to all three Ba/Ca records. Correlations were therefore performed with a significantly smaller sample size, however, contrary to precipitation, correlations using annual data were significant for Eve. Indeed, Eve showed a relatively good capacity to track river discharge (r = 0.4438, p = 0.0273, N = 25), while both Anemone and the composite record C1 did not show significant results even at the 90 % level (p = 0.52, and p = 0.1209, respectively). When averaging yearly values across other starting and ending months then January to December, such as December to November, correlations results were higher for both Eve and C1 (r = 0.526, p = 0.007, N = 25, and r = 0.426, p = 0.033, N = 25, respectively)

**Fig. 2:**
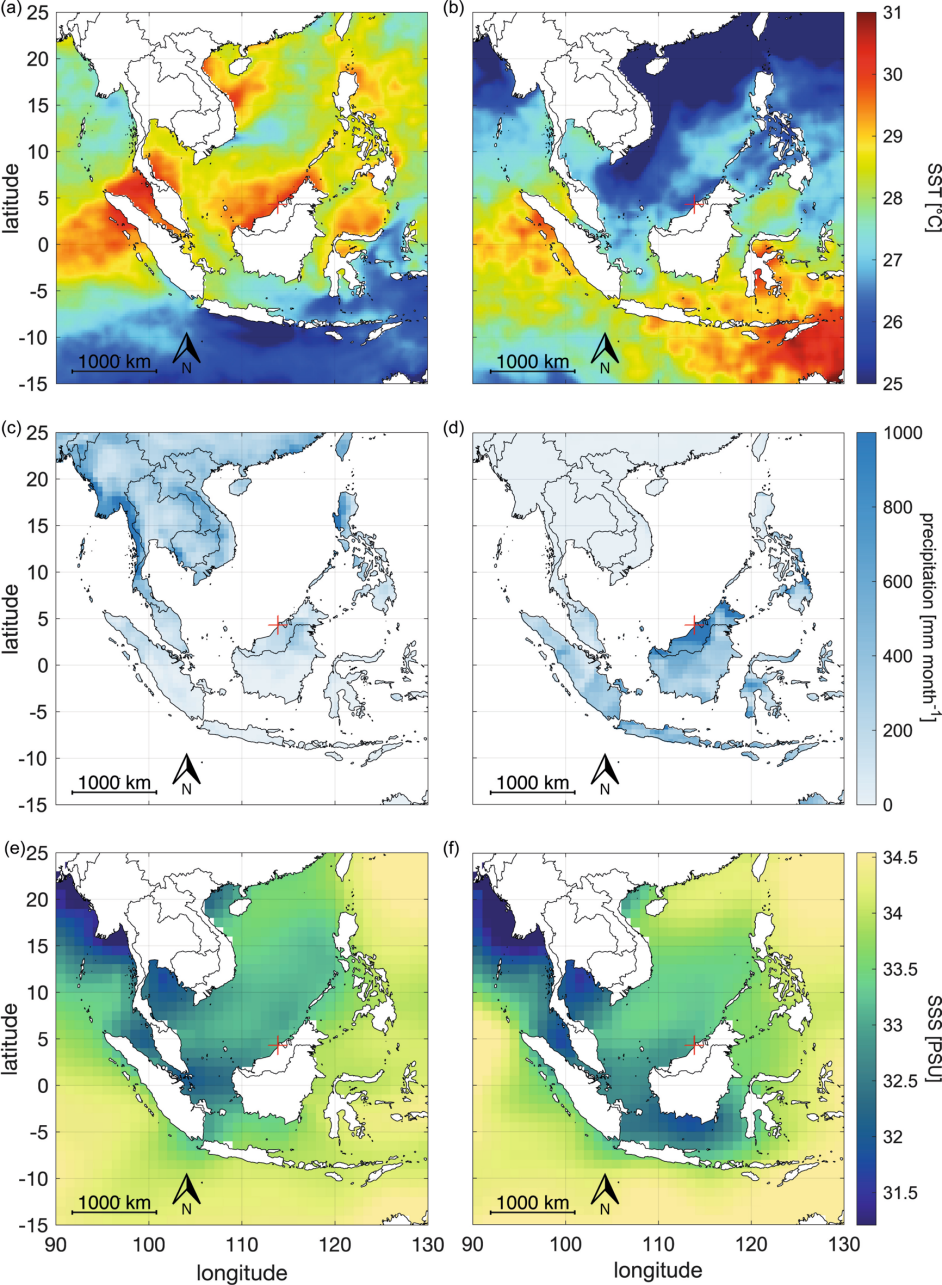
Maps of sea surface temperature, precipitation and sea surface salinity in the Maritime Continent in August during the dry season (**a, c, e**), and in January during the wet season (**b, d, f**) respectively highlighting the contrast between monsoon seasons.

To assess if the monsoon wet and dry seasons (Fig. 2) and their associated switch in annual wind and current direction had an effect on our coral proxies’ ability to record sediment in river discharge, we correlated monsoonal river discharge data with Ba/Ca.

The strengths of the winter monsoonal correlations were either comparable to annual correlations (Eve) or greater (C1: Table 1, Fig. S2). Summer monsoon records also revealed weak, non-significant correlations for all three records.

**Table 1:** Correlations of Ba/Ca records Anemone, Eve and C1 with river discharge instrumental records across both summer (June to August) and winter (February to April) monsoons. Nonsignificant correlations to the 95% level are shown in italics.

Variable	Monsoon	Ba/Ca		
		Anemone	Eve	C1
River discharge	Summer	*r* = *-0.24, p* = *0.2409, N* = *26*	*r* = *0.09, p* = *0.6480, N* = *26*	*r* =−*0.03, p* = *0.8949, N* = *26*
	Winter	*r* = *0.08, p* = *0.6864, N* = *26*	r = 0.47, p = 0.0143, N = 26	r = 0.45, p = 0.0217, N = 26

### Trend and changepoint analyses

We performed a trend analysis on the entirety of the precipitation and all three Ba/Ca records. As mentioned previously, because of the decreased data quality around 1994, the trend analysis of the Siwa record should be interpreted with caution. We used standardised data to make the comparison of each record’s slope easier.

**Fig. 3:**
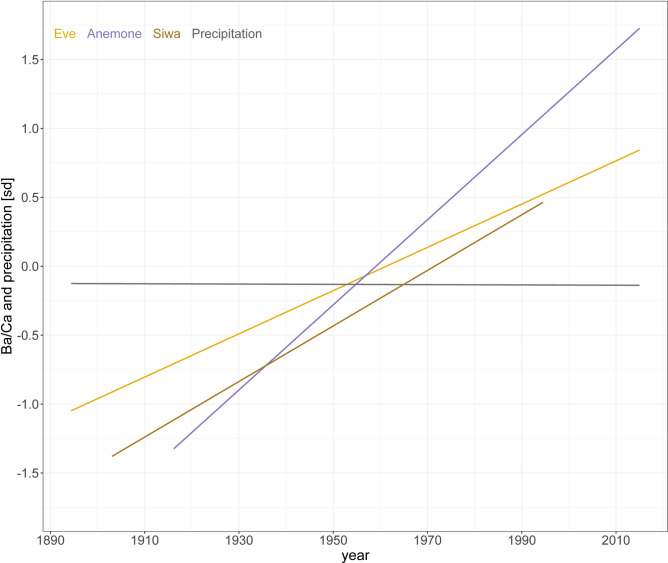
Trends in the standardised Ba/Ca record of Anemone (in blue), Eve (in yellow), Siwa (in brown) as well as precipitation (in grey) using Sen’s non–parametric method. All results show p < 0.05 except precipitation.

Although all three cores showed positive trends, Anemone’s was stronger, while the weak negative trend in the precipitation record was not significant (Fig. 3). The trend for Anemone (0.03, p < 0.001) was almost twice as strong as that of Eve (0.017, p < 0.001) and Siwa (0.02, p < 0.001), although records do not share the same length. Even when performed on Eve and Siwa starting in 1916 (the start date of Anemone), values are similar to the previous results (0.018, p < 0.001 and 0.026, p < 0.001, for Eve and Siwa, respectively), highlighting the robustness of the trend.

After establishing the presence of a positive trend amongst all Ba/Ca records, we subjected each to a changepoint analysis to define the position in time of a significant change in the arithmetic mean (decrease or increase).

**Fig. 4:**
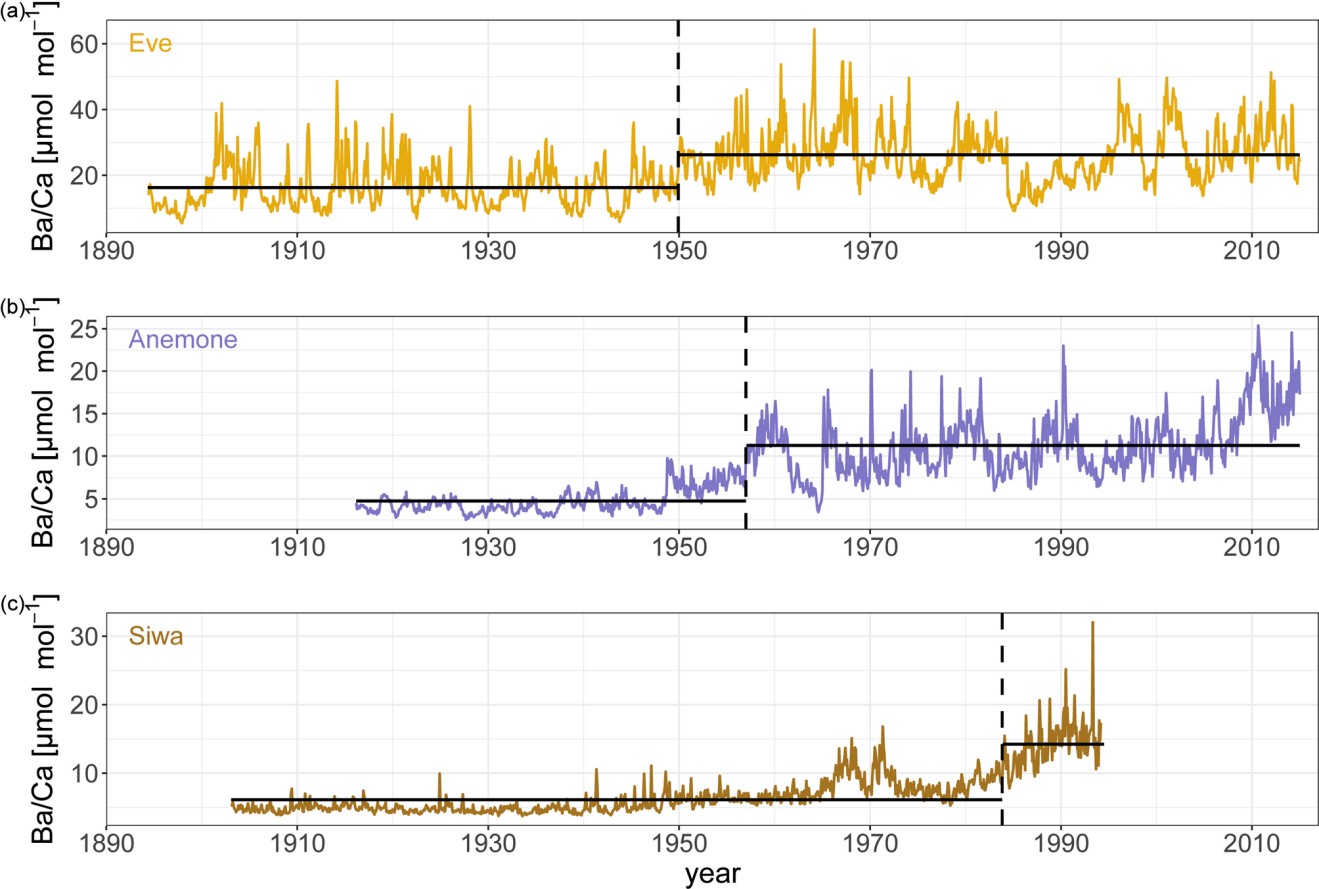
Changepoint analysis (MATLAB, Release 2023b) based on significant arithmetic mean change for (**a**) Eve, (**b**) Anemone and (**c**) Siwa.

Results show that all three records did show a significant changepoint to the 5% level separating two periods, with higher values in more recent decades (Fig. 4). The changepoint in Eve’s record occurred in December 1949, whereas the inflection in Anemone and Siwa’s records occurred in January 1957 and November 1983, respectively. In addition to showing different changepoint onsets, the mean value increases were different. Eve showed an increase of 61% in the 1950 to 2015 period over the values recorded from 1894 to 1949, while Anemone showed a 136% increase, and Siwa a 232% increase.

To confirm that the changepoint locations were not impacted by record length, we performed the analyses on Eve and Siwa’s records again after shortening their record start date to match that of Anemone. Results were identical (Fig. S3). In a similar fashion, to refute the notion that any increases in sediment delivery to the reefs are attributable to increased rainfall, we performed a changepoint analysis on the precipitation record. As expected, after showing a non-significant trend, the precipitation record showed no significant changepoint. In order to confirm that this lack of changepoint was not specific to the grid used, we also performed the same analysis on data extracted from the entirety of the area of the Baram River catchment (2.75–4.5° N, 114–115° E), and results were identical (Fig. S4). We performed the same analysis with standard deviation instead of arithmetic mean as a metric to establish if changes in the mean Ba/Ca occurred together with increased variability. The standard deviation changepoint occurred in 1950 for Eve, 1948 for Anemone, and 1964 for Siwa.

### Deforestation

The forestry data extracted from archives only provides two snapshots of timber and fuel extraction volumes for the years 1955 and 1961 across a region of the Sarawak division (4th) called “Miri”^29^. As the map only defined the 4^th^ division, we assume that the Miri region is delimited by the line joining the coast with the southward inflection of the 4th division’s limit above 3° N.

**Fig. 5:**
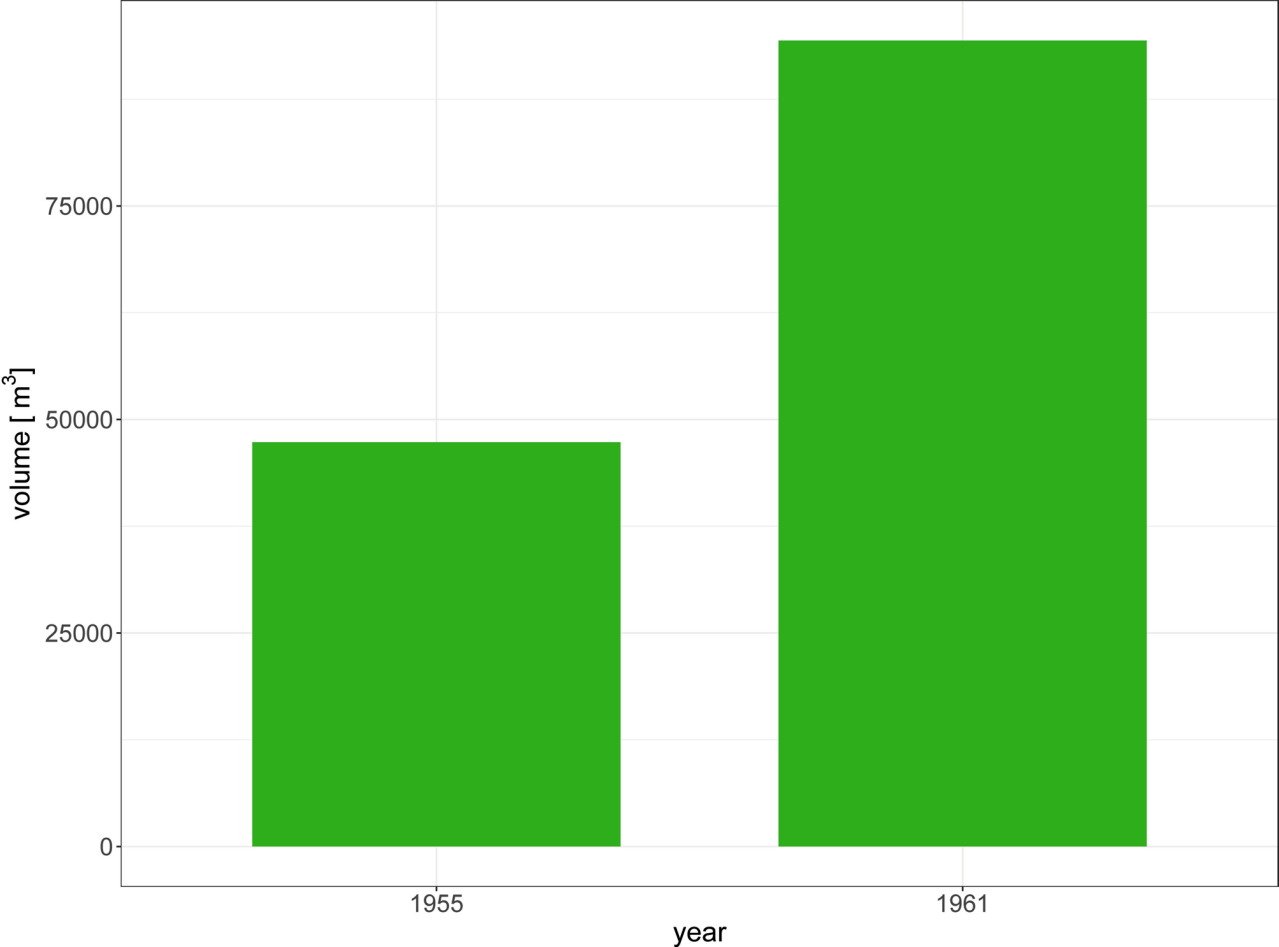
Bar plot showing the outturn of timber and fuel extraction from the Miri area in Malaysian Borneo for the years 1955 and 1961. Data extracted from Browne^29^.

Despite our assumptions, these data can still be related to deforestation as timber and fuel extractions are likely from local forest logging. These data show a doubling in the total extracted volume between 1955 and 1961 (Fig. 5).

**Fig. 6:**
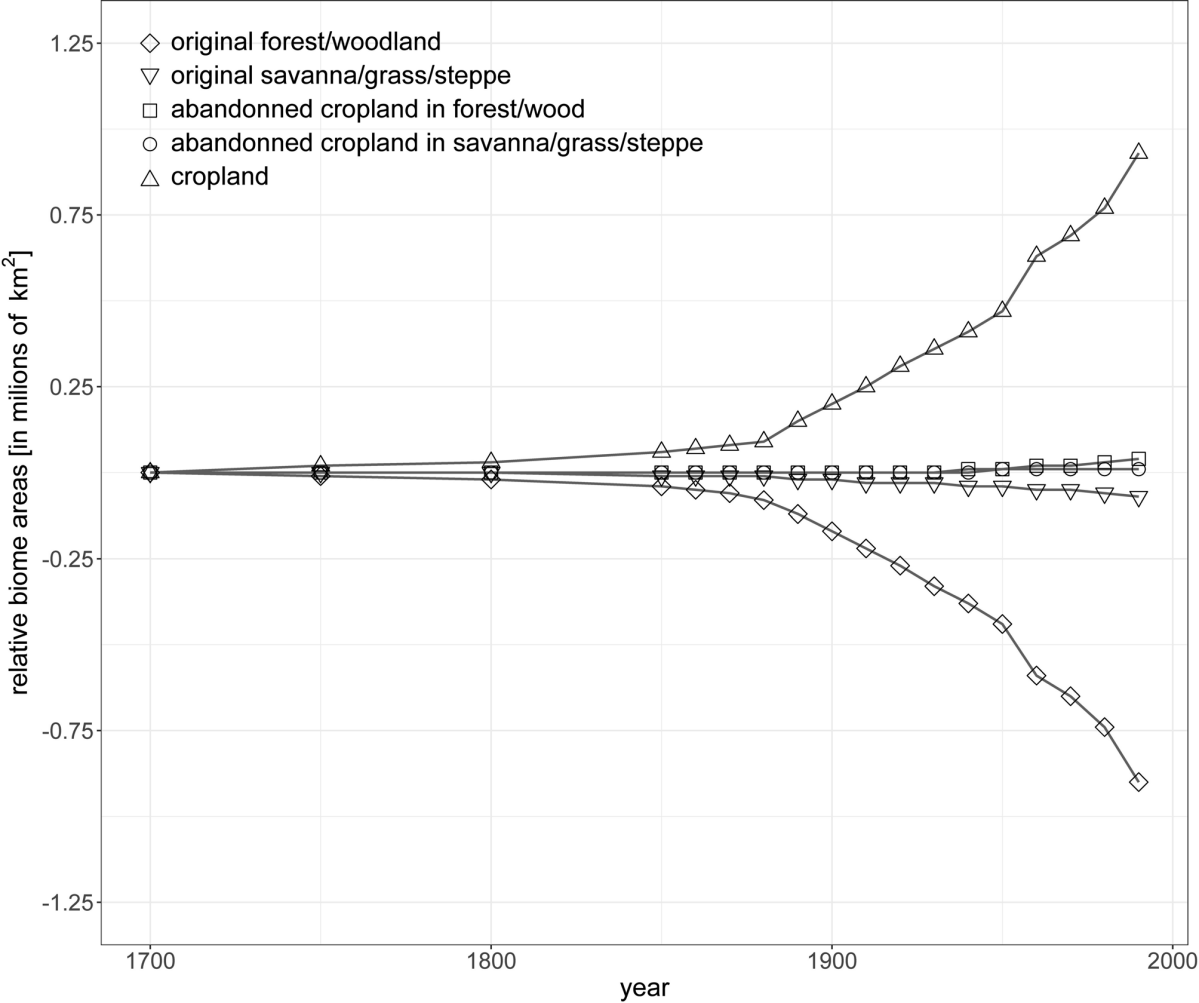
Estimated historical changes in natural vegetation extent due to clearing for croplands in Southeast Asia. Changes in areas are presented relative to the 1700 area. Diamonds represent original forest or woodland, inverted triangles represent original savanna, grass or steppe, cubes represent abandoned croplands initially in forest or woodland biomes, circles represent abandoned cropland initially in savanna, grass or steppe and triangles represent croplands. Data extracted from Ramankutty and Foley^30^.

Because these data only provide a very localised snapshot in time of actual deforestation, we also assessed the estimated land use across the entirety of Southeast Asia from 1700 to 1990 (Fig. 5) extracted from Ramankutty and Foley^30^. This data suggests that deforestation across Southeast Asia started in the middle of the nineteenth century. Indeed, the period between 1850 and 1900 shows a pronounced original forest/woodland slope of m = –0.0026 while the 1900 to 1950 period highlights an accelerated conversion of forest/woodland biomes demonstrated by a slope more than twice as steep (m = –0.0054) as the one characterising the previous period (Fig. 6). Whilst “abandoned cropland” shows very little increase or decrease, “cropland” areas increase faster than forest biome areas decreased.

## Discussion

Here, in agreement with results from a study based on the Great Barrier Reef^31^ and results from our previous study^18^, the Ba/Ca average values in each coral record showed an expected gradient coherent with the distance from the main input of Ba in the coastal environment: the Baram River mouth. However, the differences in average Ba/Ca values between adjacent colonies (Eve and Anemone, Anemone and Siwa) were not coherent with the distance from the river mouth to Anemone compared to the distance to Eve. The same can be said for Siwa when compared with Anemone or Eve. This difference points towards non-linear dissolution of Ba in the coastal waters, which is highly dependent on the mixing of water masses with different salinities and Ba concentrations^32^, as well as other processes such as adsorption/desorption to/from suspended particles^33^, or precipitation^34^. As such, the Ba concentration in coral skeletons, and thus, in surrounding seawater cannot be accurately predicted based on distance from the input source alone without extensive hydrological modelling, even if monthly riverine Ba records were available.

Conversely to the results from the initial Ba/Ca study^18^, which focussed on the period 1989–2015 only, our results when using the full twentieth century rainfall data indicate lower relationships overall, indeed, only one of our records was significantly correlated with the precipitation record across their whole length. When using shortened records (i.e. starting in 1950 or 1970), correlations improved, therefore attributing some of the low correlations results to unreliable precipitation data.

This may be somewhat surprising given that the available uninterrupted (due to model infilling) periods of local rain gauge data from the Miri airport station were located less than 13 km away from Eve’s site. Eve showed the most robust positive correlation with precipitation due to its distance with the river mouth (and plume during the winter season) and rain gauge. We suspect the lack of significant correlation, even when considering newer data, between Anemone and Siwa with precipitation is due to the larger distance between those reefs and the shore. Additionally, the proximity to two river mouths near Miri and further south in Sibuti may lead to inputs from other sediment-carrying rivers during other seasons. No river discharge data exist for the Sibuti river catchment, thus preventing us from excluding such a possibility. Finally, the strong, but not optimal, correlation between precipitation and river discharge (r = 0.68, p < 0.001, N = 25) might explain why a proxy for sediment in river discharge is not the optimal precipitation proxy as it explains less than 50% of the variation.

In contrast, a study on the Great Barrier Reef (GBR) that used Ba/Ca records to track terrestrial discharge and drought-breaking floods found significant correlations with precipitation^35^. We suspect the lack of such consistent correlations in Miri is due to the close proximity to two river mouths (unlike the GBR study where cores were several tens of kilometres from the nearest rivers), as well as direct exposure to the river plume during the winter monsoon season. Indeed, some studies with catchments similar to that of the Baram River’s have also found that precipitation did not scale linearly with river discharge^36,37^, thereby supporting our findings. This disconnect between precipitation and river discharge poses a significant challenge when attempting to track precipitation using coral Ba/Ca records, even if they are considered ideal proxies for river discharge.

Conversely, Eve, the closest record from the Baram River mouth showed a relatively strong capacity to record river discharge on annual scales and slightly stronger capacity when selecting winter monsoon months like February, March, and April, although autocorrelation was significant at a lag of 7. Likewise, several records showed better correlations when using the December to November period as it doesn’t interrupt the wet season as much as a January to December average. Similar results were found in a study from the GBR that included several records from coral colonies located at different distances from the river mouth^38^. Such a result and gradient does, however, contrast with a previous study by Lewis et al. showing the opposite trend in the same reef system. However, in this study, Lewis et al.^31^ only involved annual Ba/Ca peak maxima and coral colonies were influenced by river runoff to the north and south of the reefs, making comparison with our results difficult. Here, the use of a composite, annual data record does improve the signal-to-noise ratio but did not displaying the best fit with river discharge out of all records like expected. This is probably due to the fact that only Eve is recording the Baram River output while Anemone and Siwa are more likely influenced by a combination of the Baram and Miri rivers. In addition, both Anemone and Siwa may receive sediments from river discharge only during favourable conditions of wind driven river plume transport towards the reefs and/or a stronger river discharge signal with wider plume dispersal.

Using monsoon seasonal records and thereby taking into account the impact of seasonally changing currents on the direction of the river plume, we confirmed that some of our coral Ba/Ca records trace sediment in river discharge best during the winter monsoon. We expected significantly stronger correlations during the winter monsoon as river waters are pushed towards the coral colonies by north-easterly winds on a more regular basis. The similar or increased correlation strengths using winter monsoon records for both Eve and C1, when compared to annual data, as well as the stark contrast with results using the summer monsoon records, stresses the importance of looking beyond correlation strength (and significance) of non-transformed monthly or annual data^39,40^. Unsurprisingly, Eve, the closest record to the river mouth, showed higher correlations during the winter months than Anemone or C1 during the same period, perhaps pointing towards an optimal distance to best record sediments in river discharge, as suggested by Lewis et al.^31^, due to the way freshwater and ocean water mix as well as sedimentation settling rate. Based on this data alone, we have no way of confirming if the 28 km separating Eve from the river mouth is the optimal distance, or if anything shorter would increase correlations. This would require collecting additional coral records from colonies at sites other than Anemone, Eve, and Siwa to establish such a distance.

Previous studies have used Ba/Ca records to track changes in land use^22,41^, deforestation^41,42^, flood events^35,40^ and in population density and settlements^22^ in similar catchments in Madagascar, Indonesia, and the GBR. Additionally, a previous study in the Baram river catchment has linked deforestation with soil erosion^27^, while a sediment study showed stronger Ba concentration with decreasing distance from the Baram river mouth^28^. Similarly, we argue here that our Ba/Ca time series are recording an increase of river-bound sediments^18^ attributed to land use changes related to the onset of deforestation starting before the first half of the twentieth century. Indeed, all three Ba/Ca time series showed trends of increasing average values throughout their records and when shortened to match the record length of Anemone. However, comparably to coral Ba/Ca records from the Gulf of Chiriquí^40^, the scale of their trends was not similar. Contrasting with the trend analysis performed on the shorter version of Anemone and Eve records showing slopes of 0.067 and 0.072, respectively^18^, results here showed weaker trends, indicating a nonlinear increase of Ba/Ca throughout the records with a stronger increase towards more recent periods^43^. The difference in trend strengths between Anemone, Eve, and Siwa noted here reinforces our previous study’s hypothesis that either rates of change do not have any relationship with distance from the river mouth, or there are additional factors at play, related to local hydrodynamics that cannot be accounted for in the scope of this study^18^. We also observed an increasing lag in the position of the changepoint in time with increasing distance from the main source of Ba input (the Baram River). This pattern could potentially be due to a threshold effect related to local hydrodynamical conditions that prevented the riverine Ba signal from reaching Anemone and Siwa in amounts proportional to the distance from the river mouth^43^. Such a threshold could mean that a 61% increase in riverine Ba input was recorded by the Eve core but either was not recorded by the Anemone coral or was recorded as a lesser increase than the 28.5% greater distance from the river mouth would suggest^43^. Our results from the three coral colonies are consistent with the second option, as records further away from the river mouth need a stronger riverine Ba increase in the river discharge output to record a significant increase in their Ba/Ca signature. Further, the percentage of increased average value associated with the changepoint increased with distance from the river mouth (Fig. 3). We argue here that the mean value difference is due (at least in part) to the substantially lower baseline values found in Anemone and Siwa compared to Eve.

As such, whether the recorded increase in Ba/Ca is due to increased concentrations of sediment in river runoff or increased river discharge, or both, is currently unknown. However, for the period of overlap with instrumental data starting in 1989, we have indications for increased river runoff as a major driver of increased Ba delivery over the recent decades^18^, as our seawater Ba/Ca monitoring data show higher Ba/Ca export at nearshore sites compared to offshore reef sites (Fig. S5).

Our objective was to interpret the drivers of enhanced sediments in river discharge in the mid-20^th^ century as recorded by increased Ba/Ca baseline values across all three studied reef locations. In the absence of reliable deforestation and land-use data extending beyond the mid-1970s, we turned to historical archive data of forest logging. Such data from the Miri region in Sarawak from 1955 and 1961 shows an increase consistent with the findings of our geochemical records^29^. Although such data can hardly be transformed to approximate the deforested area without making several significant assumptions, a 100 % increase in logged timber is indicative of increased deforestation in the area, even over the short time scale between the two data points (6 years). Such a short timescale does not allow for timber wood regrowth, which has previously been observed to take between 12 to 15 years^44^. Comparably, estimated land use data across the Southeast Asia region showed a decrease in forested areas corresponding to increased deforestation and land conversion starting as early as 1850, and accelerating in the 1900s and 1950s^30^. We argue that just before 1950, while deforestation was ramping up across the Maritime Continent^30,45^, the increased sediment load resulting from deforestation in the catchment reached the threshold required to prompt a change in the Ba/Ca ratio recorded by coral colonies. The inferences we draw from geochemical data align with historical records indicating that approximately one-third of Southeast Asia’s forested areas had been cleared prior to World War II, and that this trend continued with only a gradual decline in forest cover post-1950^46^. Even though deforestation is mainly driven by commercial wood extraction, cultivation, livestock grazing, or infrastructure development in most tropical regions globally, Southeast Asia’s deforestation was primarily driven by timber logging as well as swidden cultivation and permanent agriculture during the 20^th^ century^46^. By the mid-1980s, over one-third of South-East Asia’s forests had been cleared, contrasting with South America where rapid increases in deforestation occurred earlier in the 1900s. Forest clearing in Southeast Asia does, however, show similar trends and beginnings with forests in Africa where the extraction of exotic timber was started by European settlers around 1600^46^. Although the scale of deforestation was not comparable to that undertaken in the 20^th^ century, these results indicate that by the turn of century the forested area had already been significantly reduced, contradicting previous notions of the extent of forested areas^47^.

It is imperative that we identify the onset times of deforestation to better understand rates of decline in forested areas worldwide, but particularly in tropical areas where deforestation is the most intense^1,12,48^. These efforts are essential to understand the myriads of effects that deforestation has on local and regional land and coastal ecosystems aided by a quantified changepoint corresponding to an onset date of massive deforestation. As such, results like ours should encourage further research on the impact of deforestation and land use in Malaysian Borneo had on coral reef organisms’ health to better develop science-based policy guidelines that would reduce deforestation and protect remaining ecosystems. Curbing rates of deforestation and encouraging afforestation would also reduce flood risks and enhance water quality in the coastal environment following reductions in sediment loads entering river waters^49,50^. Finally, the methods employed here should be applied to other catchments where historical instrumental data is lacking to understand the impact of anthropogenic development and uncover deforestation onsets in other tropical coastal regions.

## Conclusion

In this study, we applied previously tried-and-tested coral Ba/Ca ratios as proxies for sediment in river waters using three coral colonies from reefs increasing in distance from a major river mouth to uncover the onset of deforestation in Sarawak, Malaysian Borneo. We first assessed the relationship of Ba/Ca with river discharge across annual and monsoonal scales to quantify the impact of the monsoonal current direction switch on our coral records’ ability to track sediment in river waters. We found that corals’ Ba/Ca records showed strong correlations with river discharge during the winter monsoon season when currents direct the river plume towards the coral colonies. These results stress the importance of looking beyond simplistic monthly or annual relationships between variables when using proxy records like these. Results showed stronger correlations using a composite record in most cases, highlighting the necessity of multiple coral Ba/Ca records within and across several locations to accurately track river discharge. All three records showed low baseline values in the early 1900s (or before, in the case of Eve’s Garden) and lower values with increasing distance from the river mouth, as well as significant increases in the mean values and standard deviations, starting from 1950 and onwards. We found that Ba/Ca baseline values increased later in the twentieth century with increasing distance from the river mouth, confirming the previously theorised threshold effect. Based on earlier results showing the ability of Ba/Ca records to track river discharge, we argue that increases from 1950 and onwards are due to the onset of industrial deforestation in this region. Historical logging and estimated land use data reinforce our findings and confirm already accelerating forest logging in the Miri region from 1955, as well as deforestation as early as 1850, and accelerating deforestation between 1900 and 1950 that occurred across the wider Southeast Asia region. Our results highlight the extended length of time during which Miri’s coastal environment has been impacted by land use and deforestation and should be used as an argument in favour of limiting deforestation in the Sarawak region and across similar catchments in Borneo.

## Methods

### Coral sampling and treatment

**Fig. 7:**
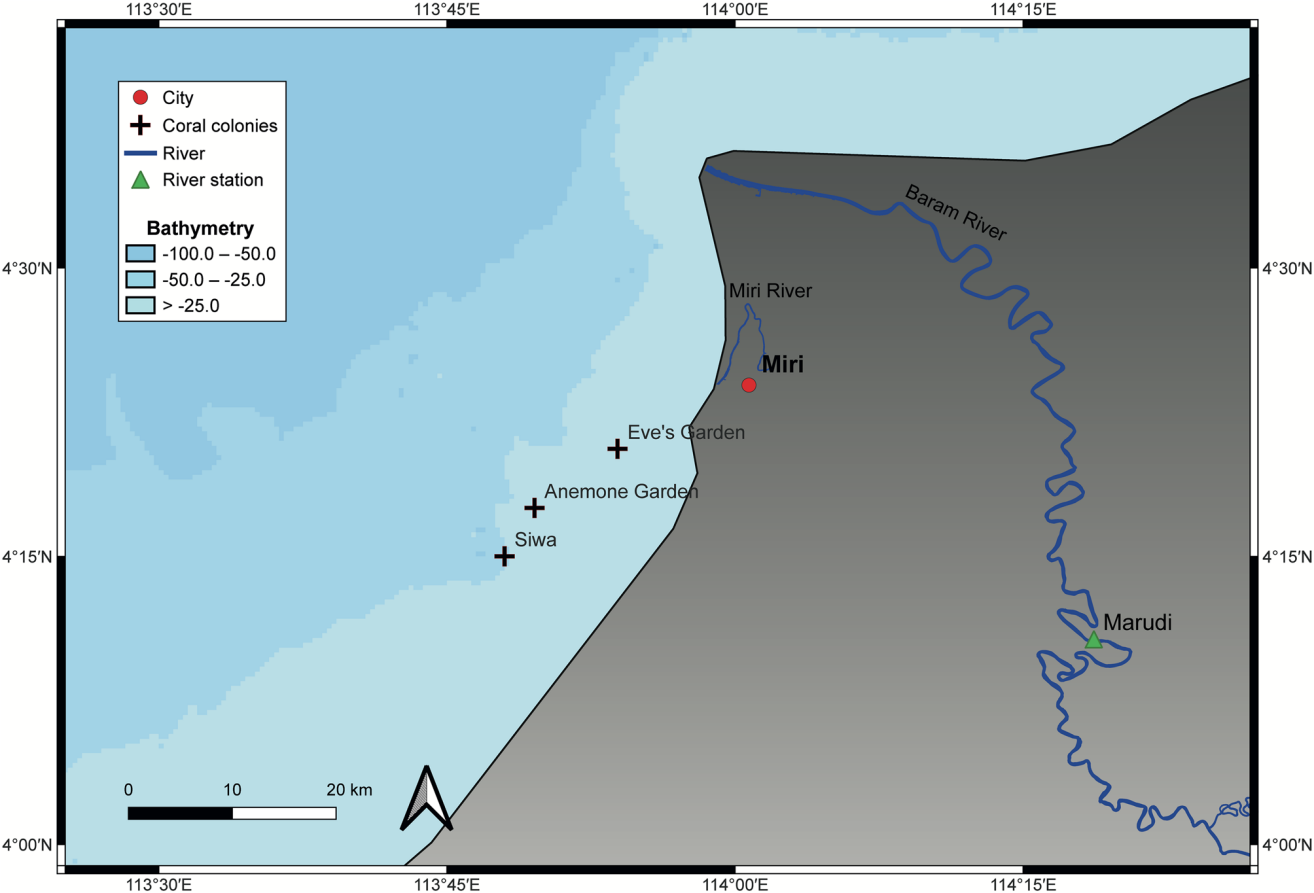
Map of the study site, the city of Miri is indicated by a red dot, coral colonies by black crosses, the Marudi river station by a green triangle and bathymetry in shades of blue.

We retrieved coral cores from Eve, Anemone and Siwa, all located within the Miri–Sibuti Coral Reefs National Park in Sarawak, Malaysian Borneo in May 2017. These three sites are located within an inshore to offshore gradient with Eve found at 5 m depth (4.34° N, 113.898° E), Anemone at 8 m depth (4.292° N, 113.826° E), and Siwa at 8 m depth (4.25° N, 113.8° E), see Fig. 7. Depths are measured from the colony’s top. We used a SCUBA air tank-driven pneumatic drill (Silverline air drill reversible) connected to a diamond-coated drill head to core through each colony. We extracted three cores, one from Eve, one from Anemone, and one from Siwa measuring 108, 93, and 114 cm, respectively.

To prepare them for sampling, cores were sectioned along the longitudinal axis into 7–8 mm thick and 4 cm wide slabs before being cleaned to ensure no organic tissue was left. We, therefore, bleached samples for 24 h in a reagent-grade sodium hypochlorite solution (NaClO, 6–14% active chlorine) bath diluted to a 1:1 ratio with deionised water (15 MΩ cm^–1^) at room temperature following a pre-existing protocol^51^. We subsequently rinsed each slab in an ultrasonic bath of deionised water (15 MΩ cm^–1^) at room temperature for ten minutes across three iterations, changing the water between each bath. Finally, we left each slab to dry in an oven set at 50 °C for 48 h. To assess what the optimal sampling path was, we used X-ray images of each slab to establish a sampling line closest to the growth axis.

### Regional setting

Our study site in the southern South China Sea (SCS) is located within the Maritime Continent separating the Indian Ocean from the Pacific Ocean. The temperature and hydroclimate in this region are influenced by the East Asian Monsoon, separating each year into a dry season (summer monsoon starting in May and ending in September), two inter-monsoon seasons (April and October) and a wet season (winter monsoon in November to March), see Fig. 2. During the dry season, the onset of south westerly winds leads to higher air and sea temperatures, weaker precipitation, and therefore higher salinities, contrasting with the wet season when north easterly winds lead to colder air and sea temperatures, stronger precipitation and lower salinities^52,53^. The East Asian monsoon drives the seasonality in this region and sea surface temperature (SST) show a 2.4 °C range between the hottest (May) and coldest (Feb) months, while rainfall ranges by 186 mm between the wettest (Dec) and driest (March) months of the year (data from 1894 to 2017). However, rainfall monthly averages display high standard deviations across all seasons and strong interannual variability^18,52,53^.

The study sites are located within 42 km of the mouth of the largest river in the area, notably the Baram River, with the smaller Miri River, opening 21.5 km to the south. The former shows a relatively large (approximate 30%) difference in values between the lowest (August) and highest (December) discharge months, where only the highest discharge month matches with the highest rainfall month. Previous studies have found this river to influence both study sites depending on the period of the year through sediment–carrying freshwater input^18,19^. Salinity, influenced by both precipitation and river discharge, exhibits a relatively small amplitude throughout the year, reaching a minimum in November and a maximum in March. It also demonstrates a better fit with the seasonality of rainfall than river discharge.

### Climate data

We obtained river discharge data for the Baram River watershed from the Department of Irrigation and Drainage of Malaysia. Although the small Miri River is believed to also influence our study site (if only marginally), only the Baram River has a record of discharge data (1989 to 2015). We used the Marudi station (4.17° N, 114.31° E) discharge data^53^ as it provides the most accurate data from a station closest to the Baram River mouth. We used the Global Precipitation Climatology Centre (GPCC) monthly precipitation data (1891 to 2015) version 2022 extracted from NOAA’s website, because of its model–filled observation-based gridded data^54^. We used the 0.25° resolution grid over 4.25–4.5° N, 113.75–114° E grid as it represents our study site the best and correlated very well with local rainfall gauge data in addition to filling the gaps found in the local data. We downloaded the data from the KNMI Climate Explorer website^55^.

### Forestry archive data

To approximate the area of deforestation that occurred before the era of satellite imagery, we extracted historical forestry data from a manuscript published online^29^, specifically, data contained in two tables from unpublished annual reports (“Colony of Sarawak. Annual Report on the Forest Department for the Year 1955” and “Colony of Sarawak. Annual Report on the Forest Department for the Year 1961”). There, each table displayed the quantities of naturally durable hardwood, other timbers, and fuel extracted from each region of Sarawak (Malaysian Borneo) in 1955 and 1961. Some values were in cubic tons while others were in hoppus tons. When totals were in hoppus tons, we converted them into m^3^ using the factor: 1 hoppus ton = 50 hoppus feet = 1.8027 m^3^. The 1955 starting date did not match our geochemical record length. We therefore used additional estimated land use data spanning 1700 to 1990 across the whole of Southeast Asia (Vietnam, Laos, Thailand, Cambodia, Malaysia, Singapore, Indonesia, Brunei, and the Philippines). For details on the quantification of land use across Southeast Asia, we refer the reader to Ramankutty and Foley^30^. These data were found and extracted from Table 3b in Ramankutty and Foley^30^, and no further data manipulation was necessary.

### Ba/Ca data

Laser ablation inductively coupled plasma mass spectrometry (LA–ICP/MS) was performed at the GeoHistory Facility in the John de Laeter Centre, Curtin University in Perth, Australia. Coral slabs were ablated using a RESOlution–SE 193 nm excimer laser equipped with a large format Laurin Technic S155 sample cell typically holding 3–4 coral slabs up to 10 cm in length, along with NIST 610/612/614^56,57^ and MACS–3^58^ standard reference materials. Optimal sampling paths were selected beforehand according to the growth axis, and analysis followed a path as close as possible to the pre–selected route. Laser fluence was calibrated above the sample cell using a hand–held energy metre and subsequent analyses were performed in constant energy mode. The cell was flushed with ultrahigh purity He (320 mL min^-1^) and N2 (1.2 mL min^-1^). High–purity argon was used as the ICP/MS carrier gas (∼1 L min^-1^). Standards and samples were ablated in line scans using an adjustable, rotating rectangular slit aperture set to 325 × 50 microns (width x length). To additionally clean the sample surface, a pre–ablation line was run at a 10 Hz laser repetition rate and 50% spot overlap before sample data acquisition. Sample ablation was then performed at a 20 μm min^-1^ scan speed, 10 Hz laser repetition rate, and on–sample laser energy of 3.2 J cm^-2^. Individual coral slabs were ablated in single continuous runs of up to 90 min, bracketed by shorter ablations (∼2 min) of the reference materials using identical laser parameters. We performed all measurements using an Agilent 7700 quadrupole ICP/MS. Each analytical session consisted of initial gas flow and ICP–MS ion lens tuning for sensitivity and robust plasma conditions (238U/232Th ∼1; 206Pb/238U ∼ 0.2; and 238UO/238U < 0.004). Pulse–analogue (P/A) conversion factors were determined on the NIST 610 reference glass by varying laser spot sizes and/or laser repetition rate to yield 1–2 Mcps per element. For data acquisition, 7Li, 11B, 25 Mg, 43Ca, 55Mn, 86Sr, 89Y, 137Ba, 208Pb, and 238U were collected with dwell times of 20 ms each after 40 s of baseline acquisition. The time–resolved mass spectra were then reduced using the ’Trace Elements’ data reduction scheme in Iolite 4.3^57–59^. Whereas the primary reference materials used in this study for the correction of instrumental drift and determination of elemental concentrations were homogeneous silicate glasses NIST 610/612 for Anemone, Eve, and Siwa cores, final trace element concentrations and element/Ca ratios were additionally normalised to the matrix–matched MACS–3 pressed carbonate reference material^58^. This secondary correction was minor for Li, Mg, Mn, Sr, Y, Ba, and Pb with corrections of 0 to 6%, but more pronounced for U (10%) and B (24%). Day–to–day variation of Ba/Ca in MACS–3 relative to the primary standard NIST610 was 9.4% (2RSD; n = 12) and serves as an indicator for overall data robustness in this study.

### Age models

Age models for Ba/Ca timeseries were built using the B/Ca record’s seasonality, as Sr was far noisier, under the assumption that growth rate was linear and constant across the year. We used SST data from the ERSSTv5 dataset^60^ to match the lowest (highest) B/Ca values (Fig. S6) of the year to the highest (lowest) temperatures. We applied a linear interpolation^18^ between each anchor point previously described and another one to 12 equidistant points per year and used the sampling date (May 2017) as a starting date resulting in a monthly resolution record. This process is prone to some time scale errors varying between one and two months because of possible inaccuracies when assigning the hottest and coldest months to minima and maxima B/Ca values as these can vary on a yearly basis and we used an average across the entirety of the record. Using this technique, we built a 123 (Eve), a 99 (Anemone), and a 114-year long (Siwa) record of Ba/Ca spanning from May 2017 to May 1894, February 1916, and February 1903, respectively.

### Data transformation

Data were standardised by subtracting the mean of the common period of the records involved (here 1916 to 2015) from each value before dividing them by their standard deviation over the same period. Both Eve and Anemone cores were used to build a composite for correlations by averaging their values across their common period (1916 to 2015), as Siwa’s record stopped in 1994. Monsoonal records of each river discharge and Ba/Ca ratios were created by averaging monthly values from February to March (winter monsoon) and from June to August (summer monsoon).

### Statistical analyses

To test for normality, we used the Kolmogorov–Smirnov test in MATLAB. Several variables were not normality distributed, therefore, all tests used here are nonparametric unless mentioned otherwise. We performed correlation tests using Spearman’s rank correlation after checking the data for autocorrelation (e.g. Figs. S7–S9). When comparing records with different units (such as Ba/Ca and river discharge), we used standardised data. To assess the presence (or not) of a trend in our records we used the Mann–Kendall nonparametric trend test. Additionally, Theil–Sen’s method was applied to estimate the true slope of the linear trend (if present) in our records. Significance is indicated by alpha = 0.05 unless stated otherwise. When looking at the existence of a point in time where our average values increase in a record, we performed a changepoint analysis^61^. Although it assumes normality, in this case the analysis isn’t impacted by the non–normality of our variables. Because we are not using the output values as estimators for the parameters of the distribution, but only the result of the search of the changepoint^62^ we were able to use this method rather than its nonparametric counterpart. The use of parametric tests reinforces our results here.

## Supplementary Information

Supplementary Information.

## Data Availability

The coral proxy data from this publication will be archived upon publication on the public NOAA WDC data portal and will be freely accessible on their website: https://www.ncdc.noaa.gov/data-access/paleoclimatology-data/datasets. The datasets used and/or analysed during the current study are available from the corresponding author on reasonable request.

## References

[1] FAO and UNEP. *The State of the World’s Forests 2020: Forests, Biodiversity and People* (FAO and UNEP, Rome, Italy, 2020). 10.4060/ca8642en.

[2] World Bank Open Data. *World Bank Open Data* https://data.worldbank.org.

[3] Wright, S. J. Tropical forests in a changing environment. *Trends Ecol. Evol.* **20**, 553–560 (2005). 16701434 10.1016/j.tree.2005.07.009

[4] Spracklen, D. V., Baker, J. C. A., Garcia-Carreras, L. & Marsham, J. H. The effects of tropical vegetation on rainfall. *Annu. Rev. Environ. Resour.* **43**, 193–218 (2018).

[5] Smith, C., Baker, J. C. A. & Spracklen, D. V. Tropical deforestation causes large reductions in observed precipitation. *Nature* **615**, 270–275 (2023). 36859548 10.1038/s41586-022-05690-1 PMC9995269

[6] Li, Y. et al. Local cooling and warming effects of forests based on satellite observations. *Nat. Commun.* **6**, 6603 (2015). 25824529 10.1038/ncomms7603 PMC4389237

[7] Butt, E. W. et al. Amazon deforestation causes strong regional warming. *Proc. Natl. Acad. Sci.* **120**, e2309123120 (2023). 37903256 10.1073/pnas.2309123120 PMC10636322

[8] Kumagai, T. et al. Annual water balance and seasonality of evapotranspiration in a Bornean tropical rainforest. *Agric. For. Meteorol.* **128**, 81–92 (2005).

[9] Labrière, N., Locatelli, B., Laumonier, Y., Freycon, V. & Bernoux, M. Soil erosion in the humid tropics: a systematic quantitative review. *Agric. Ecosyst. Environ.* **203**, 127–139 (2015).

[10] Coe, M. T., Latrubesse, E. M., Ferreira, M. E. & Amsler, M. L. The effects of deforestation and climate variability on the streamflow of the Araguaia River, Brazil. *Biogeochemistry* **105**, 119–131 (2011).

[11] Zhao, B., Lei, H., Yang, D., Yang, S. & Santisirisomboon, J. Runoff and sediment response to deforestation in a large Southeast Asian monsoon watershed. *J. Hydrol.* **606**, 127432 (2022).

[12] Matricardi, E. A. T. et al. Long-term forest degradation surpasses deforestation in the Brazilian Amazon. *Science* **369**, 1378–1382 (2020). 32913104 10.1126/science.abb3021

[13] Miettinen, J., Shi, C. & Liew, S. C. Land cover distribution in the peatlands of Peninsular Malaysia, Sumatra and Borneo in 2015 with changes since 1990. *Glob. Ecol. Conserv.* **6**, 67–78 (2016).

[14] Gaveau, D. L. A. et al. Rapid conversions and avoided deforestation: examining four decades of industrial plantation expansion in Borneo. *Sci. Rep.* **6**, 32017 (2016). 27605501 10.1038/srep32017 PMC5015015

[15] Gaveau, D. L. A. et al. Rise and fall of forest loss and industrial plantations in Borneo (2000–2017). *Conserv. Lett.* **12**, 452 (2019).

[16] Struebig, M. J. et al. Targeted conservation to safeguard a biodiversity hotspot from climate and land-cover change. *Curr. Biol.* **25**, 372–378 (2015). 25619764 10.1016/j.cub.2014.11.067

[17] Meijaard, E. & Nijman, V. Primate hotspots on borneo: predictive value for general biodiversity and the effects of taxonomy. *Conserv. Biol.* **17**, 725–732 (2003).

[18] Naciri, W. et al. Massive corals record deforestation in Malaysian Borneo through sediments in river discharge. *Biogeosciences* **20**, 1587–1604 (2023).

[19] Browne, N., Braoun, C., McIlwain, J., Nagarajan, R. & Zinke, J. Borneo coral reefs subject to high sediment loads show evidence of resilience to various environmental stressors. *PeerJ* **7**, e7382 (2019). 31428541 10.7717/peerj.7382 PMC6698134

[20] Beck, J. W., Récy, J., Taylor, F., Edwards, R. L. & Cabioch, G. Abrupt changes in early Holocene tropical sea surface temperature derived from coral records. *Nature* **385**, 705–707 (1997).

[21] Wyndham, T., McCulloch, M., Fallon, S. & Alibert, C. High-resolution coral records of rare earth elements in coastal seawater: biogeochemical cycling and a new environmental proxy3 3Associate editor: T. *J. Shaw. Geochim. Cosmochim. Acta* **68**, 2067–2080 (2004).

[22] Maina, J. et al. Linking coral river runoff proxies with climate variability, hydrology and land-use in Madagascar catchments. *Mar. Pollut. Bull.* **64**, 2047–2059 (2012). 22853989 10.1016/j.marpolbul.2012.06.027

[23] McCulloch, M. et al. Coral record of increased sediment flux to the inner Great Barrier Reef since European settlement. *Nature* **421**, 727–730 (2003). 12610621 10.1038/nature01361

[24] Prabakaran, K., Nagarajan, R., Eswaramoorthi, S., Anandkumar, A. & Franco, F. M. Environmental significance and geochemical speciation of trace elements in Lower Baram River sediments. *Chemosphere* **219**, 933–953 (2019). 30572242 10.1016/j.chemosphere.2018.11.158

[25] Tanzil, J. T. I. et al. Multi-colony coral skeletal Ba/Ca from Singapore’s turbid urban reefs: relationship with contemporaneous in-situ seawater parameters. *Geochim. Cosmochim. Acta* **250**, 191–208 (2019).

[26] Chen, X., Deng, W., Wei, G. & McCulloch, M. Terrestrial Signature in Coral Ba/Ca, δ18O, and δ13C Records From a Macrotide-Dominated Nearshore Reef Environment, Kimberley Region of Northwestern Australia. *J. Geophys. Res. Biogeosciences* **125**, e2019JG005394 (2020).

[27] Vijith, H., Seling, L. W. & Dodge-Wan, D. Estimation of soil loss and identification of erosion risk zones in a forested region in Sarawak, Malaysia, Northern Borneo. *Environ. Dev. Sustain.* **20**, 1365–1384 (2018).

[28] Nagarajan, R. *et al.* Chapter 12 - Geochemical Characterization of Beach Sediments of Miri, NW Borneo, SE Asia: Implications on Provenance, Weathering Intensity, and Assessment of Coastal Environmental Status. In *Coastal Zone Management* (eds. Ramkumar, Mu., James, R. A., Menier, D. & Kumaraswamy, K.) 279–330 (Elsevier, 2019). 10.1016/B978-0-12-814350-6.00012-4.

[29] Browne, F. G. *Sarawak: Miscellaneous Reports* (1956).

[30] Ramankutty, N. & Foley, J. A. Estimating historical changes in global land cover: Croplands from 1700 to 1992. *Glob. Biogeochem. Cycles* **13**, 997–1027 (1999).

[31] Lewis, S. E. et al. A critical evaluation of coral Ba/Ca, Mn/Ca and Y/Ca ratios as indicators of terrestrial input: new data from the Great Barrier Reef. *Australia. Geochim. Cosmochim. Acta* **237**, 131–154 (2018).

[32] Santos, I. R. et al. Uranium and barium cycling in a salt wedge subterranean estuary: the influence of tidal pumping. *Chem. Geol.* **287**, 114–123 (2011).

[33] Hanor, J. S. & Chan, L.-H. Non-conservative behavior of barium during mixing of Mississippi River and Gulf of Mexico waters. *Earth Planet. Sci. Lett.* **37**, 242–250 (1977).

[34] Bishop, J. K. B. The barite-opal-organic carbon association in oceanic particulate matter. *Nature* **332**, 341–343 (1988).

[35] Saha, N. et al. Seasonal to decadal scale influence of environmental drivers on Ba/Ca and Y/Ca in coral aragonite from the southern Great Barrier Reef. *Sci. Total Environ.* **639**, 1099–1109 (2018). 29929279 10.1016/j.scitotenv.2018.05.156

[36] Li, H., Luo, L., Wood, E. F. & Schaake, J. The role of initial conditions and forcing uncertainties in seasonal hydrologic forecasting. *J. Geophys. Res. Atmos.* **114**, 745 (2009).

[37] Ivancic, T. J. & Shaw, S. B. Examining why trends in very heavy precipitation should not be mistaken for trends in very high river discharge. *Clim. Change* **133**, 681–693 (2015).

[38] Jupiter, S. et al. Linkages between coral assemblages and coral proxies of terrestrial exposure along a cross-shelf gradient on the southern Great Barrier Reef. *Coral Reefs* **27**, 887–903 (2008).

[39] Pfeiffer, M. & Dullo, W.-C. Monsoon-induced cooling of the western equatorial Indian Ocean as recorded in coral oxygen isotope records from the Seychelles covering the period of 1840–1994AD. *Quat. Sci. Rev.* **25**, 993–1009 (2006).

[40] Brenner, L. et al. Evaluating the reproducibility and paleo-hydroclimate potential of coral skeletal Ba/Ca in the Gulf of Chiriquí, Panama. *Cont. Shelf Res.* **267**, 105104 (2023).

[41] Fleitmann, D. et al. East African soil erosion recorded in a 300 year old coral colony from Kenya. *Geophys. Res. Lett.* **34**, 145 (2007).

[42] Hartmann, A. et al. Multi-proxy evidence for human-induced deforestation and cultivation from a late Holocene stalagmite from middle Java. *Indonesia. Chem. Geol.* **357**, 8–17 (2013).

[43] Lewis, S. E., Shields, G. A., Kamber, B. S. & Lough, J. M. A multi-trace element coral record of land-use changes in the Burdekin River catchment NE Australia. *Palaeogeogr. Palaeoclimatol. Palaeoecol.* **246**, 471–487 (2007).

[44] Okuda, S., Corpataux, L., Muthukrishnan, S. & Wei, K. H. Cross-laminated timber with renewable and fast growing tropical species in South East Asia. In *Proceedings of theWorld Conference on Timber Engineering (WCTE 2018), Seoul, Korea* 20–23 (2018).

[45] Brookfield, H. & Byron, Y. Deforestation and timber extraction in Borneo and the Malay Peninsula: The record since 1965. *Glob. Environ. Change* **1**, 42–56 (1990).

[46] Lambin, E. F. & Geist, H. J. Regional differences in tropical deforestation. *Environ. Sci. Policy Sustain. Dev.* **45**, 22–36 (2003).

[47] Aleman, J. C., Jarzyna, M. A. & Staver, A. C. Forest extent and deforestation in tropical Africa since 1900. *Nat. Ecol. Evol.* **2**, 26–33 (2018). 29230024 10.1038/s41559-017-0406-1

[48] Ordway, E. M., Asner, G. P. & Lambin, E. F. Deforestation risk due to commodity crop expansion in sub-Saharan Africa. *Environ. Res. Lett.* **12**, 044015 (2017).

[49] Schwärzel, K., Ebermann, S. & Schalling, N. Evidence of double-funneling effect of beech trees by visualization of flow pathways using dye tracer. *J. Hydrol.* **470–471**, 184–192 (2012).

[50] Duffy, C. et al. The impact of forestry as a land use on water quality outcomes: an integrated analysis. *For. Policy Econ.* **116**, 102185 (2020).

[51] Nagtegaal, R. et al. Spectral luminescence and geochemistry of coral aragonite: effects of whole-core treatment. *Chem. Geol.* **318–319**, 6–15 (2012).

[52] Tangang, F. T. & Juneng, L. Mechanisms of Malaysian Rainfall Anomalies. *J. Clim.* **17**, 3616–3622 (2004).

[53] Saadi, Z., Shahid, S., Chung, E.-S. & Ismail, T. Projection of spatial and temporal changes of rainfall in Sarawak of Borneo Island using statistical downscaling of CMIP5 models. *Atmospheric Res.* **197**, 446–460 (2017).

[54] Schneider, U., Hänsel, S., Finger, P., Rustemeier, E. & Ziese, M. GPCC Full Data Monthly Product Version 2022 at 0.25°: Monthly Land-Surface Precipitation from Rain-Gauges built on GTS-based and Historical Data. 10.5676/DWD_GPCC/FD_M_V2022_025 (2022).

[55] Trouet, V. & Van Oldenborgh, G. J. KNMI climate explorer: a web-based research tool for high-resolution paleoclimatology. *Tree-Ring Res.* **69**, 3–13 (2013).

[56] Hollocher, K. & Ruiz, J. Major and trace element determinations on nist glass standard reference materials 611, 612, 614 and 1834 by inductively coupled plasma-mass spectrometry. *Geostand. Newsl.* **19**, 27–34 (1995).

[57] Jochum, K. P. et al. GeoReM: a new geochemical database for reference materials and isotopic standards. *Geostand. Geoanalytical Res.* **29**, 333–338 (2005).

[58] Wilson, S. A., Koenig, A. E. & Orklid, R. Development of microanalytical reference material MACS-3 for LA-ICP-MS analysis of carbonate samples. *Geochim. Cosmochim. Acta* **72**(12S), A1025–A1025 (2008).

[59] Woodhead, J. D., Hellstrom, J., Hergt, J. M., Greig, A. & Maas, R. Isotopic and elemental imaging of geological materials by laser ablation inductively coupled plasma-mass spectrometry. *Geostand. Geoanal. Res.* **31**, 331–343 (2007).

[60] Huang, B. et al. Extended reconstructed sea surface temperature, version 5 (ERSSTv5): upgrades, validations, and intercomparisons. *J. Clim.* **30**, 8179–8205 (2017).

[61] MATLAB. MATLAB and Signal Processing Toolbox. The MathWorks, Inc. (Release 2023b).

[62] Trauth, M. H. *MATLAB® Recipes for Earth Sciences*. (Springer International Publishing, Cham, 2021). 10.1007/978-3-030-38441-8.

