# Supplementary material for: Corals Ba/Ca records uncover mid-twentieth century onset of land use change associated with industrial deforestation in Malaysian Borneo
Source: Sci Rep. 2025 Jul 1;15:21410. doi: 10.1038/s41598-025-06679-2 (PMC12218137; doi:10.1038/s41598-025-06679-2)
Supplement: Supplementary file 1 — Supplementary Information. [file 41598_2025_6679_MOESM1_ESM.docx]

## **Supplementary information**

| year | Forested area [x10^6^ ha] |
| --- | --- |
| 1973 | 9.224 |
| 2000 | 8.217 |
| 2005 | 7.956 |
| 2010 | 7.335 |
| 2015 | 6.837 |

**Supplementary Table 1: Forested area in the state of Sarawak, Malaysian Borneo. Data are extracted from Gaveau et al. 2014**^1^**.**

**
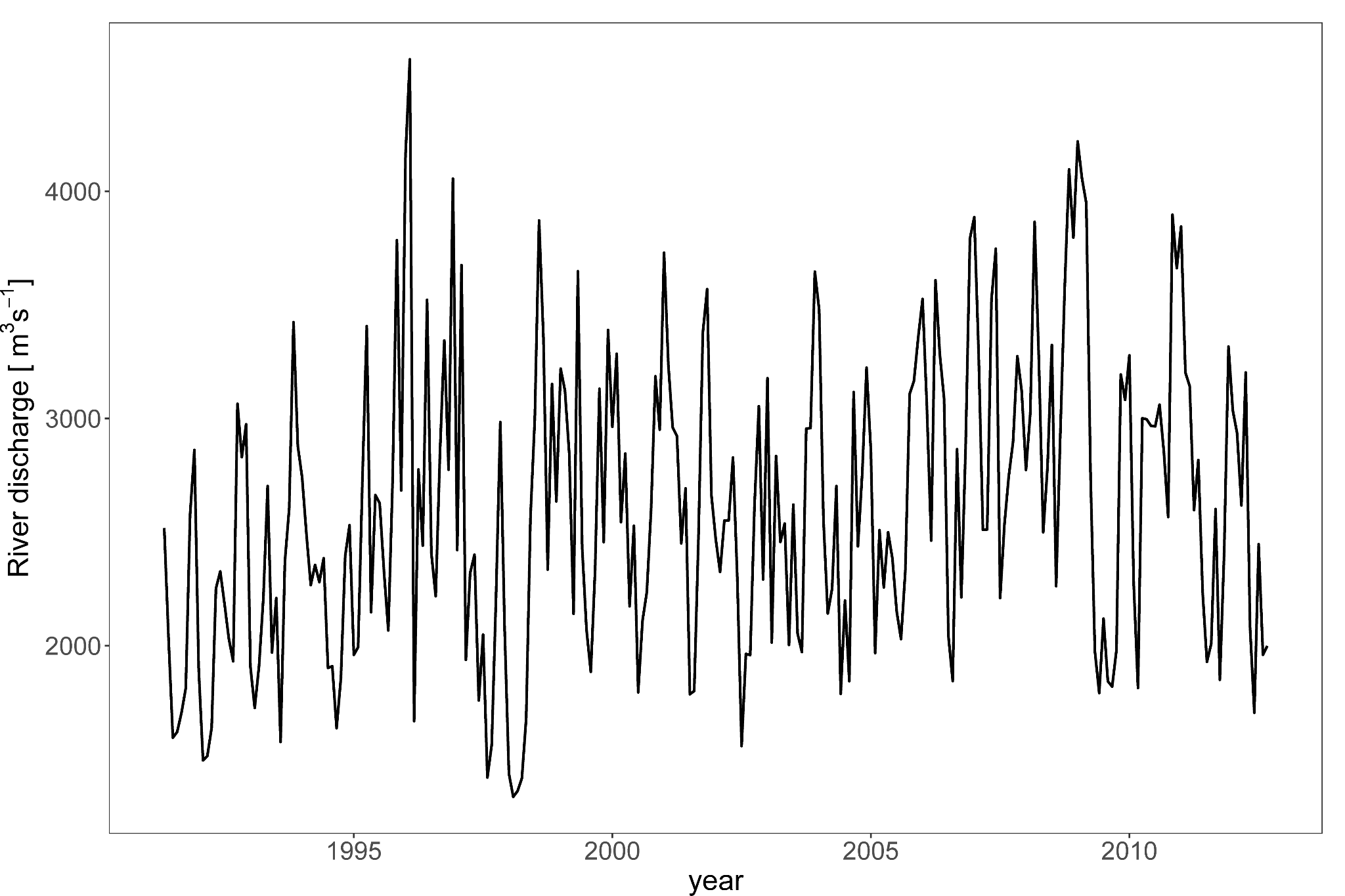
**

**Supplementary Figure 1: Monthly record of the Baram River discharge based on the flow at the Marudi station (4.178° N, 114.311° E).**

**
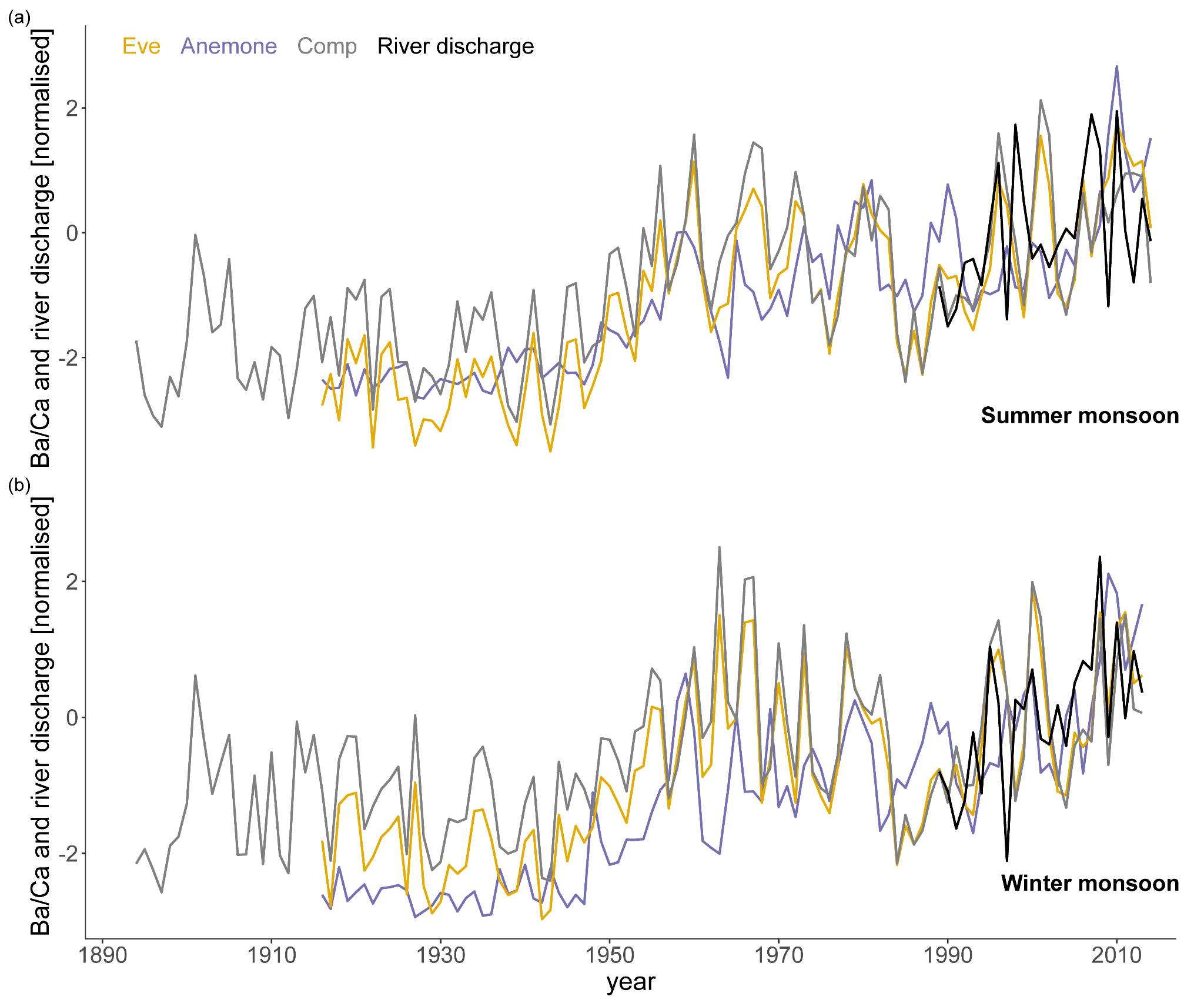
**

**Supplementary Figure 2: Annual Ba/Ca records of Eve, Anemone and the composite (in yellow, blue and grey, respectively) as well as river discharge (in black) during the summer (a) and winter (b) monsoon.**

**
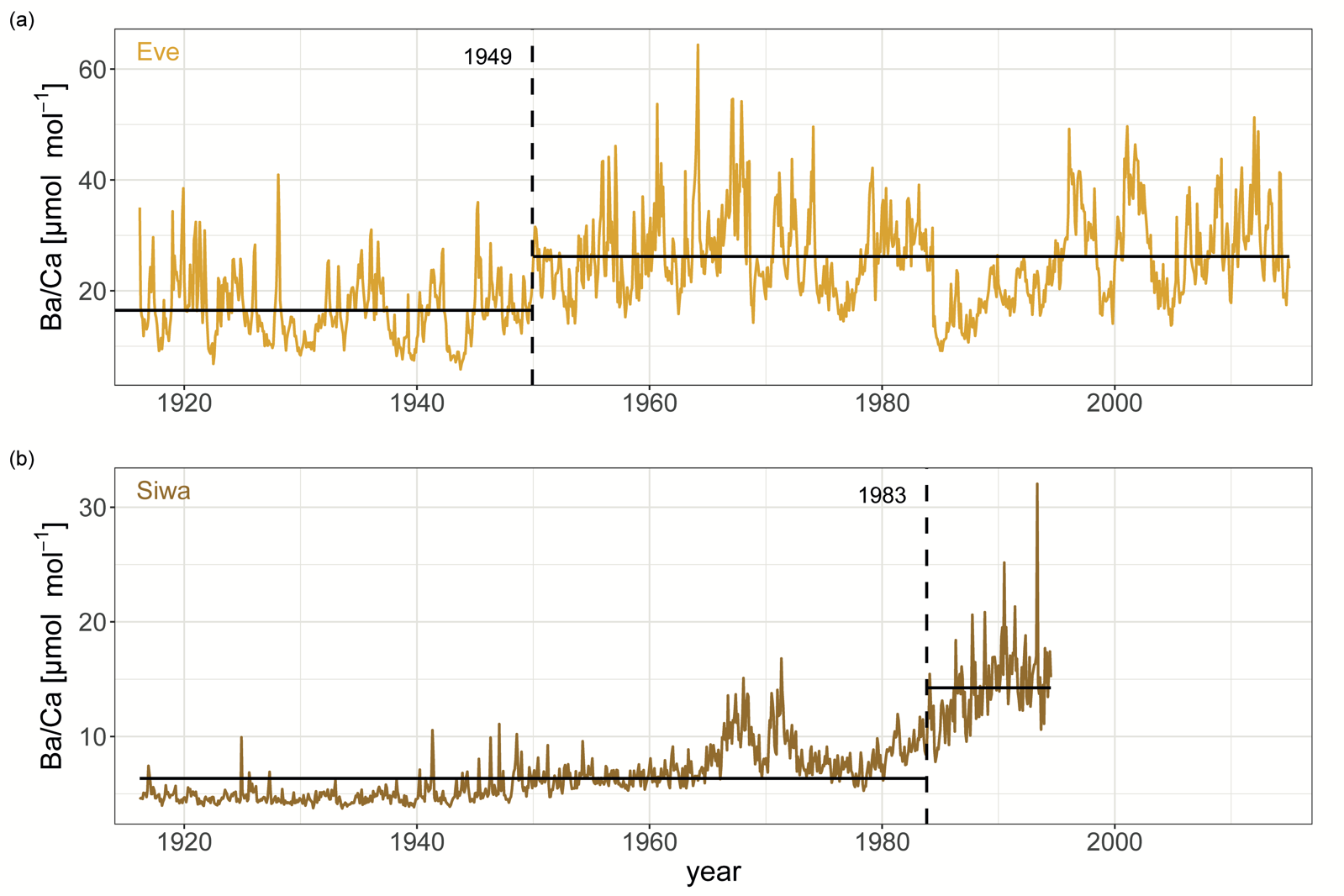
Supplementary Figure 3: Changepoint analysis**^2^ **based on significant arithmetic mean change for (a) Eve and (b) Siwa between 1916 and 2015, and 1916 and 1994, respectively.**

**
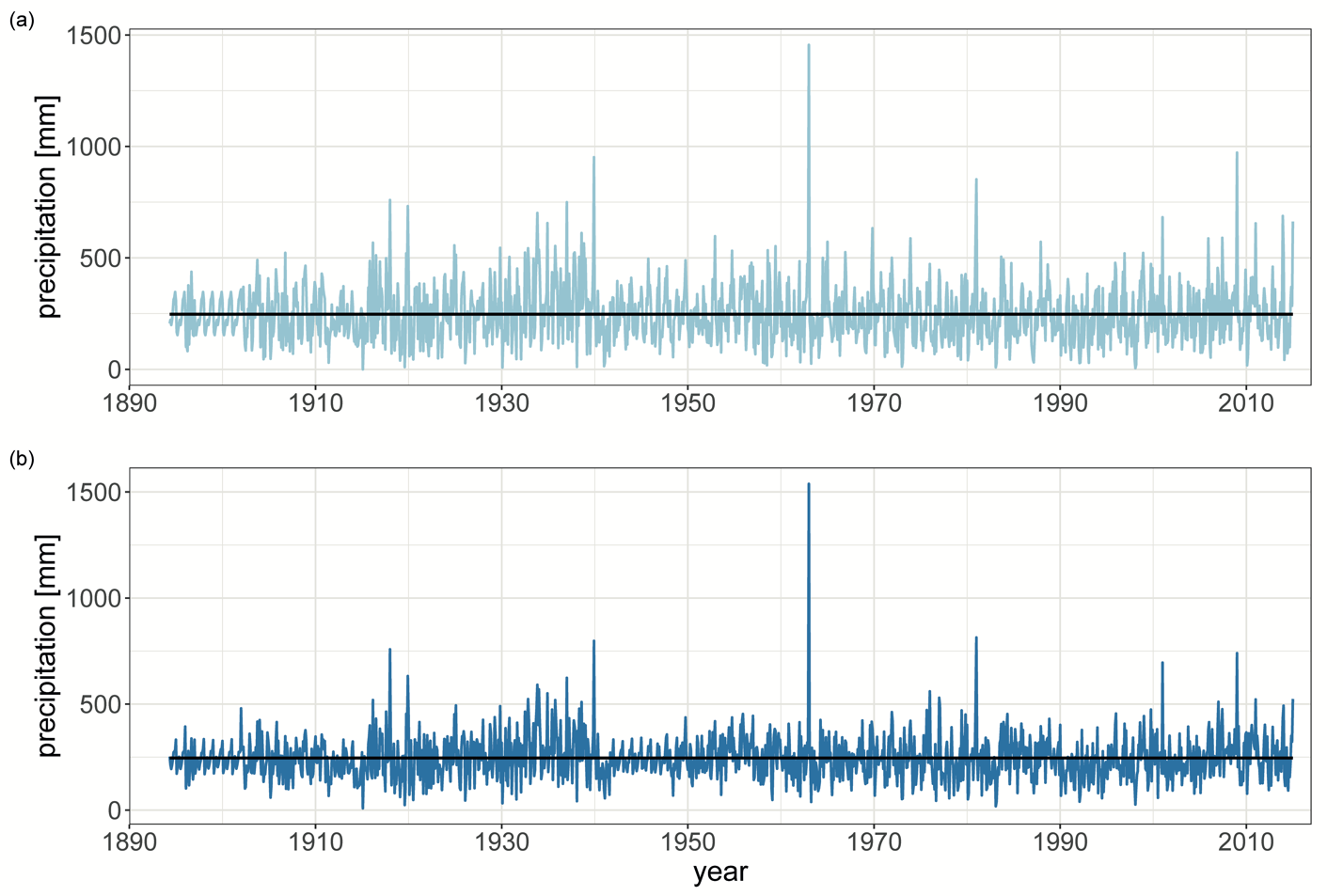
**

**Supplementary Figure 4: Changepoint analysis**^2^ **based on significant arithmetic mean change displaying no significant change across the record for rainfall (a) at our study site (4-5° N, 113­-114° E) and (b) for the whole Baram river catchment (2.75-4.5° N, 114-115° E).**


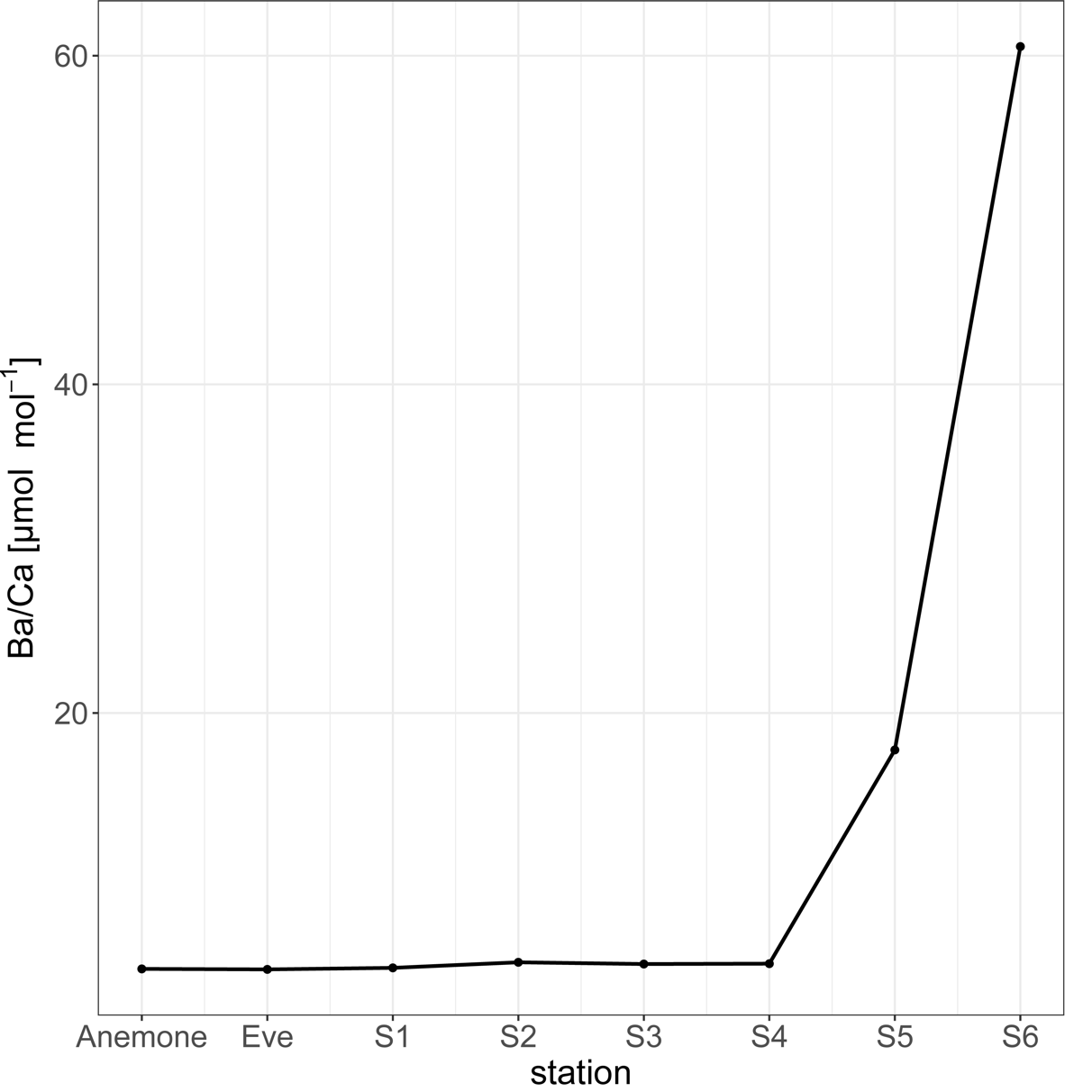


**Supplementary Figure 5: Ba/Ca values of seawater across a transect from Anemone and Eve and the Miri River located approximately 11 and 21 km away from Eve’s Garden and Anemone’s Garden, respectively. Samples were obtained from the surface (top 10 cm) on October 11^th^, 2019, during the monsoon transition season.**

**
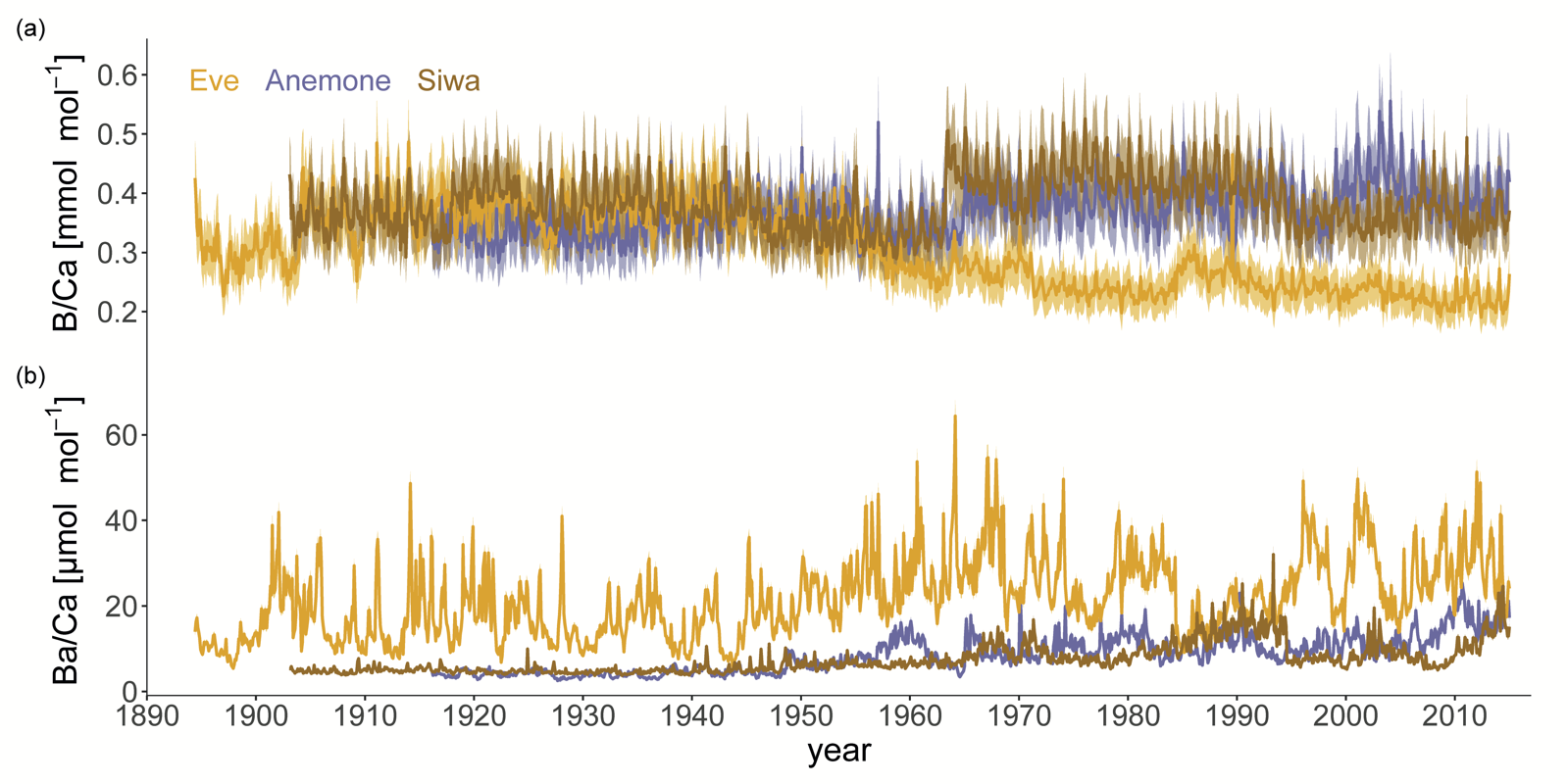
**

**Supplementary Figure 6: Monthly interpolated time series of (a) B/Ca and (b) Ba/Ca for Even in yellow, Anemone in blue, and Siwa in brown. Shading indicates analytical uncertainty.**


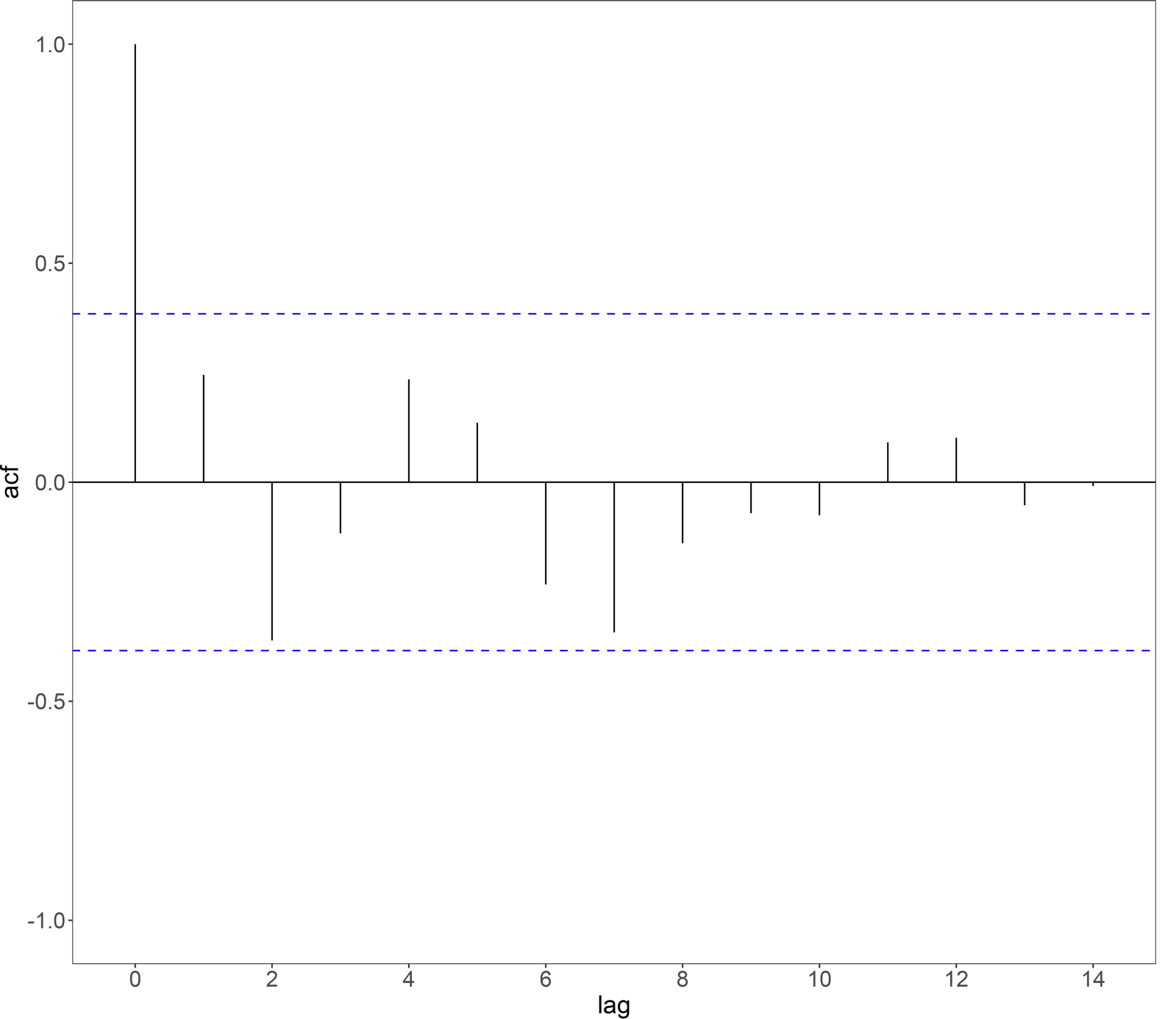


**Supplementary Figure 7: Autocorrelation function of the winter monsoonal Eve record. Blue lines indicate 95 % confidence interval.**


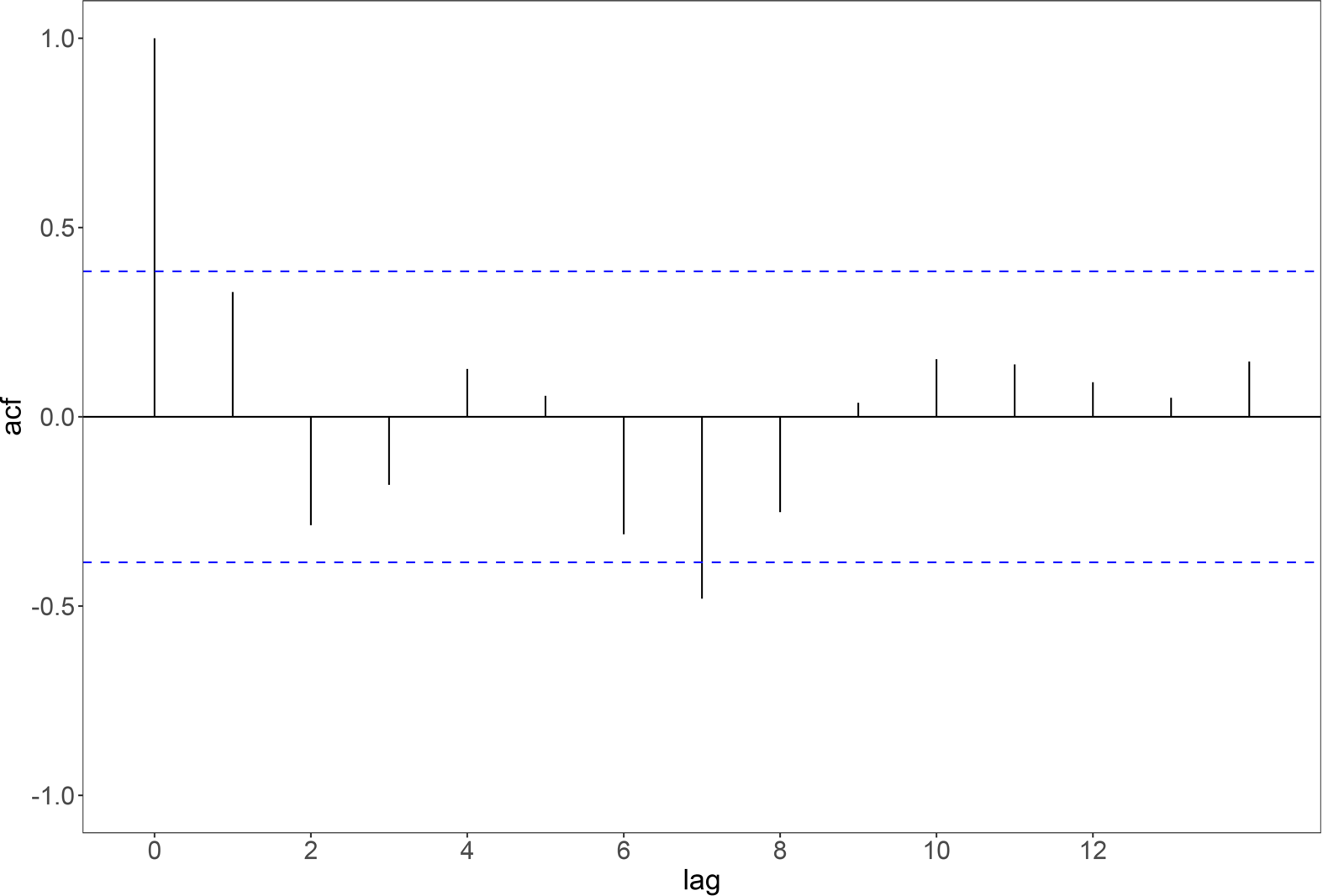


**Supplementary Figure 8: Autocorrelation function of the winter monsoonal C1 record. Blue lines indicate 95 % confidence interval.**


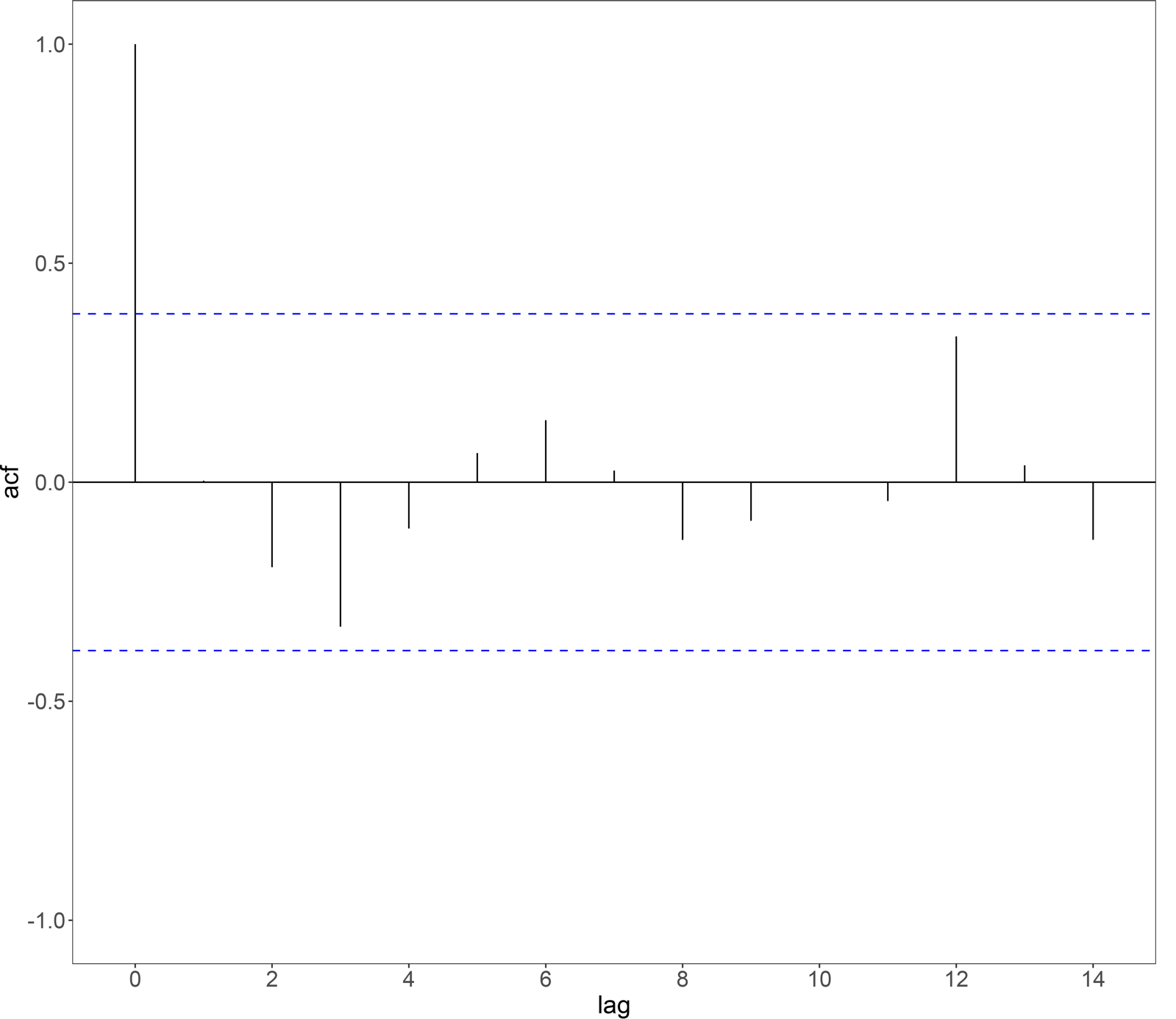


**Supplementary Figure 9: Autocorrelation function of the winter monsoonal river discharge record. Blue lines indicate 95 % confidence interval.**

## **References**

1. Gaveau, D. L. A. *et al.* Four Decades of Forest Persistence, Clearance and Logging on Borneo. *PLoS ONE* **9**, e101654 (2014).

2. MATLAB. MATLAB and Signal Processing Toolbox. The MathWorks, Inc. (Release 2023b).
